# Real-time jute leaf disease classification using an explainable lightweight CNN via a supervised and semi-supervised self-training approach

**DOI:** 10.3389/fpls.2025.1647177

**Published:** 2025-10-24

**Authors:** Meftahul Jannat, Md Shahab Uddin, Mohammad Asif Hasan, Md Saimun Alam, Avijit Paul, Muhammad E. H. Chowdhury, Julfikar Haider

**Affiliations:** ^1^ Department of Electronics and Telecommunication Engineering, Rajshahi University of Engineering and Technology, Rajshahi, Bangladesh; ^2^ Department of Computer Science, Maharishi International University, Fairfield, IA, United States; ^3^ Department of Electrical and Electronic Engineering, Chittagong University of Engineering and Technology, Chittagong, Bangladesh; ^4^ Department of Electrical Engineering, Qatar University, Doha, Qatar; ^5^ Department of Engineering, Manchester Metropolitan University, Manchester, United Kingdom

**Keywords:** deep learning, semi-supervised self-training, lightweight CNN, grouped convolution, squeeze-and-excite, jute leaf disease

## Abstract

**Introduction:**

Timely detection of jute leaf diseases is vital for sustaining crop health and farmer livelihoods. Existing deep learning approaches often rely on large, annotated datasets, which are costly and time-consuming to produce.

**Methods and results:**

To address this challenge, a lightweight convolutional neural network integrated with a semi-supervised learning self-training framework was proposed to enable accurate classification with minimal labeled data. The model combines modified depthwise separable convolutions, an enhanced squeeze-and-excite block, and a modified mobile inverted bottleneck convolution block, achieving strong representational power with only 2.24M parameters (8.54 MB). On a self-collected dataset of jute leaf images across three classes (Cescospora leaf spot, golden mosaic, and healthy leaf), the proposed model achieved a best accuracy of 98.95% under the supervised training with training, testing and validation split of 80:10:10. Remarkably, the model also attained a best accuracy of 97.89% in the semi-supervised learning (SSL) setting with only 10% labeled and 90% unlabeled data, demonstrating that near-supervised performance can be maintained while substantially reducing the dependency on costly labeled datasets. The application of explainable AI method such as Grad-CAM provided interpretable visualizations of diseased regions, and deployment as a Flask-based web application demonstrated practical, real-time usability in resource-constrained agricultural environments.

**Conclusion:**

These results highlight the novelty of combining SSL with a lightweight CNN to deliver near-supervised performance, improved interpretability, and real-world applicability while substantially reducing the dependence on expert-labeled data.

## 1 Introduction

Jute, often known as the “golden fiber,” is a vital crop because of its importance in the biodegradable and eco-friendly natural fiber industry. It is cultivated primarily in parts of Bangladesh and India, particularly in the Ganges Delta, where it thrives in the region’s warm, humid climate and fertile alluvial soil. Other major producers include China, Thailand, Myanmar, Indonesia, Brazil, and Nepal, highlighting its global economic importance (Mahapatra et al., 2009). Bangladesh is currently the world’s second-largest producer of jute, accounting for 42% of global production, which amounts to 1.33 million tons (Yasmin and Moniruzzaman, 2024). One of its most notable advantages is its biodegradability and renewability, making it a sustainable alternative to synthetic fibers and a key player in efforts to reduce plastic pollution (Miah et al., 2011). Widely used in industries such as textiles, packaging, and agriculture, jute offers an eco-conscious substitute for plastic-based materials, decomposing naturally without leaving harmful residues. In addition to its environmental benefits, jute cultivation supports millions of smallholder farmers, providing them with a stable source of income while also generating employment opportunities in processing, manufacturing, and trade. As a result, jute not only contributes to sustainability but also strengthens rural economies, reinforcing its status as a valuable global commodity.

However, the productivity and quality of jute are persistently threatened by a spectrum of diseases, such as stem rot (Macrophomina phaseolina (Ghosh et al., 2018)), root rot (Rhizoctonia solani (Wadud and Ahmed, 1962)), Cercospora leaf spot (Sarkar and Gawande, 2016), golden mosaic (Biswas et al., 2013), and viral infections such as the jute mosaic virus (Hasan et al., 2015). These diseases not only lead to major economic losses for farmers but also exacerbate the hardships faced by smallholder communities that rely on jute cultivation for their livelihood. The impact of these infections can extend beyond the farm, affecting industries dependent on jute-based products and disrupting supply chains. The impact of jute mosaic disease on jute production has been studied, and it is recognized as a major constraint to successful jute cultivation (Ghosh et al., 2012). Therefore, timely detection and accurate identification of these diseases are critical for implementing effective management strategies, such as disease-resistant crop varieties, improved farming practices, and targeted biological or chemical treatments. Proactive measures and continuous research on plant pathology and disease control are essential for safeguarding jute production, ensuring both economic stability for farmers and the long-term sustainability of this vital crop.

Traditional methods for disease detection largely rely on manual inspection and expert knowledge, which are often time-consuming, subjective, and impractical for large-scale monitoring. Moreover, limited access to expert pathologists in remote farming regions hampers timely intervention. In this context, the advent of machine learning (ML), Deep Learning (DL) and computer vision offers a transformative avenue for automating and enhancing disease detection processes (Ye et al., 2019; Wijethunga et al., 2023). While supervised learning models, particularly CNNs (Haque et al., 2024), have shown promise in plant disease recognition, they demand extensive labeled datasets to achieve high accuracy a luxury not always available for jute diseases owing to insufficient annotated images and variability in symptom expression. This scarcity of labeled data poses a significant hurdle in developing robust diagnostic models (Zhao et al., 2022).

To address these challenges, this research explores a semi-supervised learning approach that leverages the abundance of unlabeled jute plant images alongside a limited set of labeled examples to enhance disease detection and classification (Learning, 2006). Semi-supervised learning techniques are particularly well-suited for agricultural applications, where labeled data are often scarce, expensive, and time-consuming to obtain. By effectively utilizing both labeled and unlabeled data, these methods can significantly improve learning accuracy and model generalizability.

The objectives of this research are threefold, each aimed at advancing the field of jute disease detection through the integration of cutting-edge ML and image processing techniques. First, the study seeks to develop a Lightweight supervised CNN model for detecting and classifying various jute diseases. Then, the same model is applied in semi-supervised learning. This model leverages both labeled and unlabeled image data to improve learning efficiency, thereby addressing the critical challenge of limited annotated datasets. By incorporating semi-supervised techniques, this research aims to reduce the dependency on extensive manual labeling while still achieving high-precision classification of jute diseases. Second, the research aims to increase the accuracy and efficiency of disease diagnosis by integrating DL architectures with advanced image processing techniques. The proposed approach employs state-of-the-art CNN and feature extraction methods to refine disease identification, ensuring early detection and timely intervention. This enhancement will contribute to minimizing crop losses and optimizing disease management strategies for farmers. Third, the study intends to develop real time application to provide agronomists and farmers with an easy solution to identify the jute leaf diseases with higher confidence in an early stage. This comprehensive evaluation ensures that the developed system is not only highly accurate in controlled settings but also practical and reliable for deployment in real agricultural environments.

This study contributes to the advancement of jute disease detection by introducing a novel lightweight DL architecture and leveraging semi-supervised learning to address the challenge of limited labeled data. The main novel contributions of this study are as follows:

1. A novel lightweight DL model was proposed using modified depthwise separable convolutions, along with an enhanced Squeeze and Excite (SE) block and a modified mobile inverted bottleneck convolution (MBconv).
2. A high-quality dataset comprising 920 meticulously categorized images of jute leaves, including three distinct classes, namely, healthy, Cercospora leaf spot, and golden mosaic, was developed for jute leaf disease classification.
3. The proposed model incorporates a semi-supervised self-training (ST) method to address the limitation of data labeling towards building a more robust jute leaf disease classification model.
4. Explainable AI tools such as Grad-CAM was used to identify the region of interest to find the diseased area more accurately.
5. A web application was developed to deploy the proposed model in a real-time agricultural setting, demonstrating its practical applicability and usability under field conditions.

The remainder of this manuscript is organized as follows: Section 2 provides a comprehensive review of related works in the domain of plant disease classification. Section 3 details the methodology, including dataset preparation, preprocessing, the proposed lightweight CNN architecture, semi-supervised self-training framework, and deployment strategies. Section 4 presents experimental results, performance analysis, complexity evaluation, and Grad-CAM-based explainability outcomes. Section 5 presents comparative analysis with the literature, the implications of the findings, strengths and limitations, and outlines potential future research directions. Finally, Section 6 concludes based on the key findings of the study.

## 2 Related works

The identification of jute leaf diseases has been significantly constrained by the limited availability of comprehensive disease-related labeled datasets. Despite this challenge, researchers have made efforts to detect diseases by existing data. To address this limitation, they developed advanced and complex models designed across various datasets to increase the accuracy and effectiveness of several plants’ leaf disease detection techniques.

Salam et al. (2024) explored the effectiveness of various transfer learning models, including MobileNetV3Small, ResNet50, VGG19 for mulberry leaf disease classification. The authors used four additional convolutional layers added to each model for modification. MobileNetV3Small outperformed the other models, achieving an accuracy of 96.4% on a dataset comprising 6,000 images across 3 mulberry leaf disease classes. This study uses explainable AI such as Grad-CAM to highlight areas influencing the decision made by the model. Additionally, the authors developed a mobile app to use in real life application. However, the absence of a dedicated large-scale dataset for mulberry leaf diseases limited the development and validation of robust detection models.

Karim et al. (2024) proposed a modified MobileNetV3large model to classify 4 types of grape leaf diseases using 27,122 leaf images achieving a 99.66% accuracy. The authors used Grad-CAM to highlight the model’s decision-making areas on the leaf images. However, the authors did not develop any mobile application to use in the real-life grape leaf disease detection.

Padhi et al. (2024) employed EfficientNetB4 with compound scaling and Swish activation on a paddy leaf disease dataset containing a total of 19,131 images with 10 classes of paddy leaf diseases. This method achieved the accuracy of 96.91%. The study incorporated diverse data augmentation techniques and rigorous evaluation metrics, providing valuable insights into model optimization. However, the authors did not use any XAI technique to display the model’s behavior to identify the diseased area on leaf.

Khan et al. (2024) introduced a Bayesian optimized multimodal deep hybrid learning approach for tomato leaf disease classification. The authors employed a custom CNN model for feature extraction, followed by seven classical machine learning classifiers including Random Forest, XGBoost, GaussianNB, SVM, MLR, KNN, and a stacking ensemble. Bayesian optimization and the Tree-structured Parzen Estimator (TPE) were used for hyperparameter tuning, and a Boruta feature selection layer was added to identify the most relevant features. The CNN-Stacking model achieved the highest performance, with 98.27% accuracy, 98.53% recall, and 98.53% precision on a dataset of 18,159 images across 10 classes of tomato leaf disease. However, the study lacks an actual mobile or desktop application deployment, and its use of a single, well-curated dataset raises concerns about generalizability to real-world, heterogeneous field data. Additionally, despite high accuracies, potential model overfitting and a need for interpretability and explainability remain areas for further improvement.

Naresh Kumar and Sakthivel (2025) proposed a novel rice leaf disease classification framework using a hybrid Fusion Vision Boosted Classifier (FVBC) that combines the VGG19 convolutional neural network for feature extraction with the LightGBM gradient boosting algorithm for classification. The FVBC model achieved high performance with training, validation, and testing accuracies of 97.78%, 97.5%, and 97.6%, respectively using a dataset comprising 2,627 images divided across six classes. Despite its strong classification results, the study does not incorporate any explainable AI (XAI) techniques to interpret the model’s decisions, nor does it include the development of a practical application such as a mobile or web interface for end-user deployment.

Bansal et al. (2024) proposed a federated CNN approach using datasets from five different clients representing various jute-growing environments. Their model classified five jute leaf disease types using 4200 images and achieved classification accuracies ranging between 79.87% to 83.67% across federated clients. However, limitations in this study include the absence of real-world deployment and explainability, which could hinder farmer trust.

Kaushik and Khurana (2025) implemented a deep learning model based on the ResNet50 architecture, fine-tuned using transfer learning for binary classification of jute leaves as either healthy or diseased. The dataset contained 1,820 images. After preprocessing and data augmentation, the model achieved a classification accuracy of 94%. While the model was computationally efficient and demonstrated strong performance, it was limited to only two classes, reducing its applicability in real-world settings where multiple disease types coexist.

Rajput et al. (2024) proposed a federated CNN architecture enhanced with decision tree support for classifying five distinct jute leaf diseases: anthracnose, stem rot, root rot, Macrophomina wilt, and yellow mosaic virus. The dataset was sourced from five clients representing various ecological regions. Performance evaluation across clients showed macro, micro, and weighted averages peaking at 94.27%, 94%, and 94.28%, respectively, with the best classification accuracy observed at 98% for one client. Although the integration of decision trees aimed to enhance interpretability, no formal XAI methods were used to explain model decisions. Additionally, there was no application interface proposed for deployment, which limits its field adoption.

Tanny et al. (2025) introduced a deep ensemble learning based model DERIENet where the features ere extracted using ResNet50, InceptionV3 and EfficientNetB0 and then best features were selected global maxpooling method and concatenated together. Using a Kaggle dataset of 920 images divided in three classes cercospora leaf spot, golden mosaic, healthy, expanded to 7,800 images via augmentation, the model achieved 99.95% accuracy with near-perfect precision, recall, and AUC scores.

Uddin and Munsi (2023) developed a CNN model with four convolutional layers, four max-pooling layers, and two fully connected layers. The dataset comprises 4740 jute leaf images, categorized into three classes: healthy, yellow mosaic, and powdery mildew. The proposed CNN model achieved a high classification accuracy of 96%, outperforming models like SVM (83.28%) and GPDCNN (93.12%). While the model’s performance is strong, the study lacks transparency in terms of model interpretability and does not explore deployment strategies, suggesting the need for further validation and explainability enhancements in future research.

Most of the aforementioned techniques rely on complex model architectures to address the challenges posed by limited labeled data. However Benchallal et al. (2024) applied semi-supervised learning to weed species classification, introducing the ConvNeXt-Base-SSL model and evaluating it on the DeepWeeds dataset with 8 classes, the 4-Weeds dataset with 4 classes, and the CIFAR-10 dataset with 60,000 images and 10 classes. Their approach achieved over 90% accuracy when using datasets where only 20% of the images were labeled. Although this research has focused primarily on weed species identification, its applicability to other image classification tasks may be limited. The method relies on a deep encoder-decoder architecture with many trainable parameters, which could be computationally expensive. Nonetheless, this work outperformed several state-of-the-art supervised models, highlighting the potential of semisupervised learning to achieve high accuracy with minimal labeled data and offering a promising direction for more efficient annotation strategies in future research.

Wang et al. (2024) presented a plant disease classification model based on self-supervised learning (SSL), integrating a Masked Autoencoder (MAE), a Convolutional Block Attention Module (CBAM), and a Gated Recurrent Unit (GRU). The authors used two datasets: a self-collected dataset consisting of 3256 images of potato leaves categorized into three classes (early blight, late blight, healthy), and the CCMT dataset with 88,010 images across 18 disease classes from crops such as tomato, maize, cassava, and cashew. The proposed method achieved high classification accuracies: 99.61% on the self-collected dataset and 95.35% on the CCMT dataset. While the model utilized advanced attention mechanisms (CBAM) and sequence processing (GRU), it did not incorporate explainable AI (XAI) techniques, and no practical application or mobile/desktop app was reported. Limitations of the paper include the absence of XAI for interpretability and no real-world deployment.

Ilsever and Baz (2024) explored consistency regularization-based semi-supervised learning for plant disease recognition, employing the Mean Teacher approach to address challenges related to limited labeled data, dataset balance, batch size, and fine-tuning strategies. Using datasets such as PlantVillage, Plant Pathology 2021, and a newly created PP2021TS with 38 classes and 54,309 images, the study implemented three fine-tuning strategies for ResNet: HeadOnly, HeadThenBody, and Mean-Teacher. Among these methods, the Mean-Teacher method achieved the highest accuracy of 88.50% when trained on just 5% labeled data, outperforming conventional supervised learning methods. Despite requiring two models (student and teacher), this approach demonstrated the effectiveness of semi-supervised learning in agricultural applications, highlighting its potential for improving plant disease detection with minimal labeled data.

While numerous studies have demonstrated the potential of supervised learning across different plant datasets, a significant gap persists in addressing the jute leaf dataset classification, where labeled data are very limited. Collecting and labeling large datasets is both time-consuming and resource-intensive. Several studies have demonstrated the efficacy of supervised learning approaches in the classification of other leaf diseases, but there remains a notable gap in the literature regarding classifying jute leaf disease via both supervised and semi-supervised approaches. Although previous studies in plant disease detection have made significant strides using deep learning and self-supervised approaches, substantial gaps remain particularly in the domain of XAI techniques such as Grad-CAM and real time mobile or web app deployment. The absence of research on jute leaf disease classification highlights an opportunity to explore methods that leverage both labeled and unlabeled data, potentially improving classification performance while reducing the burden of manual annotation.

## 3 Methodology

The subsequent sections detail the methodology employed to develop the lightweight custom CNN model for the classification of jute leaf diseases utilizing both supervised and semi-supervised learning methods. Specifically, the following section outlines the proposed semi-supervised learning framework, the custom CNN architecture developed for jute leaf disease classification, and the experimental setup used to evaluate the model’s performance.

### 3.1 Dataset description

The dataset used in this experiment consists of 920 high-quality images that have been carefully organized into three classes: Cescospora leaf spot, golden mosaic, and healthy leaf, containing 309, 347, and 264 images, respectively. These images were collected through extensive fieldwork in Dinajpur and Brahmanbaria, two significant agricultural regions in Bangladesh, with full authorization and support from local agricultural officials. The dataset was labeled with the help of agronomists who provided expert annotations to ensure the accuracy and reliability of disease classifications, thereby enhancing the overall quality and credibility of the training data (Alam, 2024). As the previous studies did not make the jute leaf datasets publicly available, a new dataset was developed for providing greater accessibility by other researchers. Furthermore, the disease classes in this dataset are different from the classes reported in the literature contributing to unique data resource development for future research.

In image classification tasks, data leakage occurs when information from the validation or test set inadvertently becomes available during training. This usually happens if augmented versions of the same original image are distributed across training, validation, and testing subsets. For example, if an original leaf image is placed in the training set, but its rotated or flipped version appears in the validation or test set, the model indirectly exposed to the same information during both training and evaluation. This leads to overly optimistic performance that do not reflect true generalization ability of the model. To mitigate this issue, for each class, the original non-augmented leaf images were split using 80:10:10 ratio, where 80% of the labeled images were used for training, 10% for validation, and the remaining 10% for testing to evaluate the performance of the model. The training images were augmented to increase the number of images needed to make a large dataset. After the augmentation, 3,000 images were selected for each class to create a balanced dataset, resulting in a total of 9,000 training images. The validation and test sets remained unaltered to provide unbiased evaluation. In **Table 1**, the data distribution for the supervised method is shown. For the semi-supervised method, 80% of the data allocated for training were further divided into two subsets: 10% served as labeled data, while the remaining 90% was treated as unlabeled. The same validation and testing sets from the supervised approach were retained to ensure consistent performance evaluation. From **Table 2**, the data distribution can be easily understood, clearly illustrating the proportions of labeled, unlabeled, validation, and testing sets used in both supervised and semi-supervised methods.

**Table 1:** Data distribution in each jute leaf disease class for supervised learning.

Class name	Original	Training	Training post augmentation	Validation	Testing
Cescospora Leaf Spot	309	247	3000	30	32
Golden Mosaic	347	277	3000	34	36
Healthy Leaf	264	212	3000	25	27

**Table 2:** Data distribution in each jute leaf disease class for semi-supervised learning.

Class name	Original	Training post augmentation	Training		Validation	Testing
			Labeled (10%)	Unlabeled (90%)		
Cescospora Leaf Spot	309	3000	300	8100	31	31
Golden Mosaic	347	3000	300		35	35
Healthy Leaf	264	3000	300		26	26

### 3.2 Dataset preprocessing and augmentation

**Figure 1:**
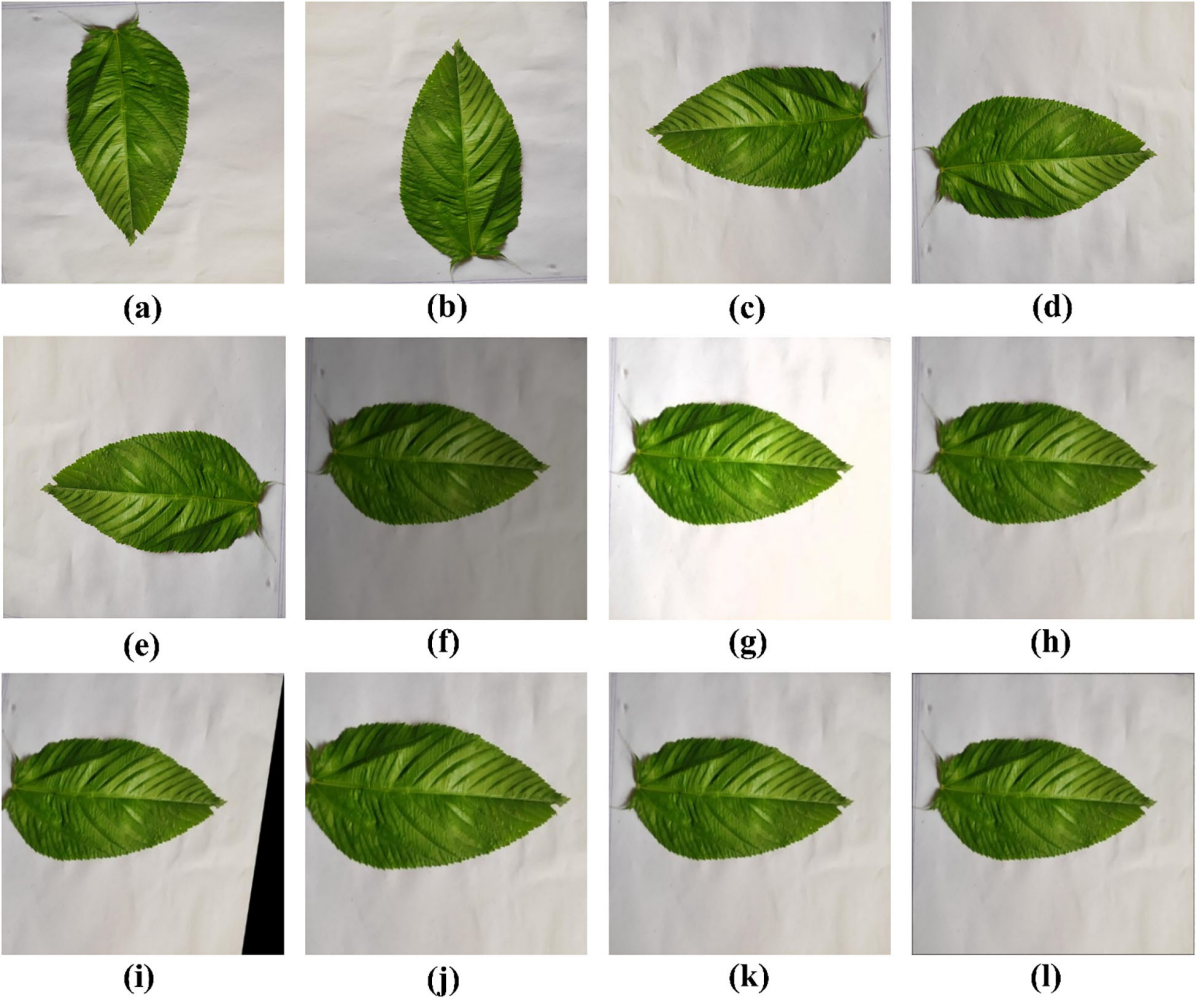
Augmented images: **(a)** rotate 90 degrees clockwise **(b)** rotate 90-degree counter-clockwise, **(c)**horizontal flip, **(d)** vertical flip, **(e)** horizontal–vertical flip, **(f)** brightness decrease, **(g)** brightness increase, **(h)** Gaussian blur, **(i)** shearing, **(j)** zoom in, **(k)** zoom out, **(l)** perspective transform.

Owing to the varying sizes of the images in the original dataset, uniform scaling was necessary as a preprocessing step. Every image was resized to 224 × 224 pixels. Images typically show a wide range of intensity levels. Normalization was used to convert the scale from 0–255 to 0–1. All the pixel values were divided by 255 to normalize the intensity level. Moreover, to increase the size of the dataset, several augmentation techniques were applied, including rotation (± 90°), horizontal and vertical flips, combined horizontal and vertical flips, brightness adjustments (increase/decrease), Gaussian blur, shearing, zooming in/out, and perspective transformation. Examples of these operations are shown in **Figure 1**.

### 3.3 Overall model architecture

**Figure 2:**
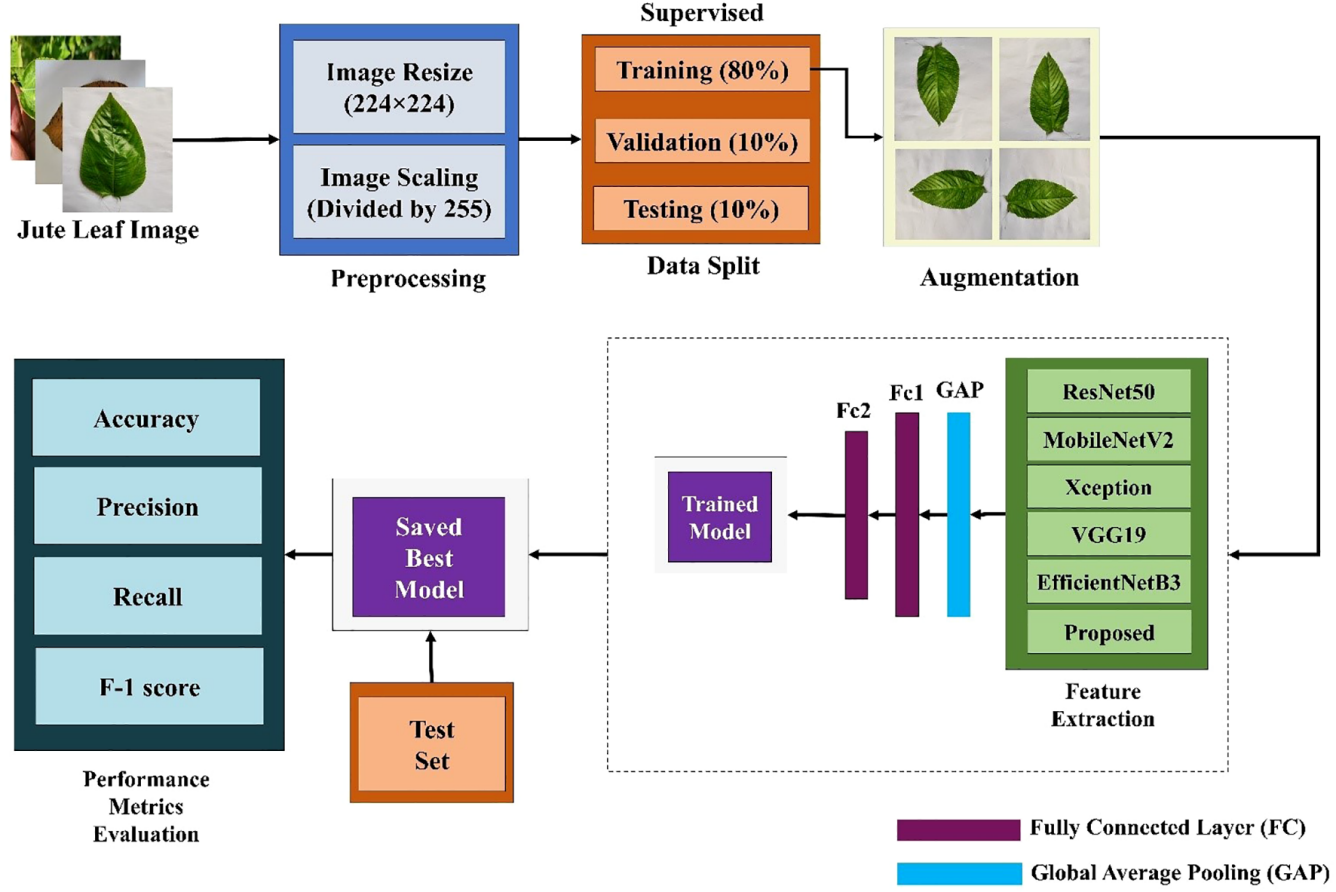
Workflow of the proposed supervised jute leaf disease classification pipeline, showing preprocessing, data augmentation, train–validation–test split (80:10:10), feature extraction using TL and the proposed CNN model, performance evaluation, and model saving.

**Figure 3:**
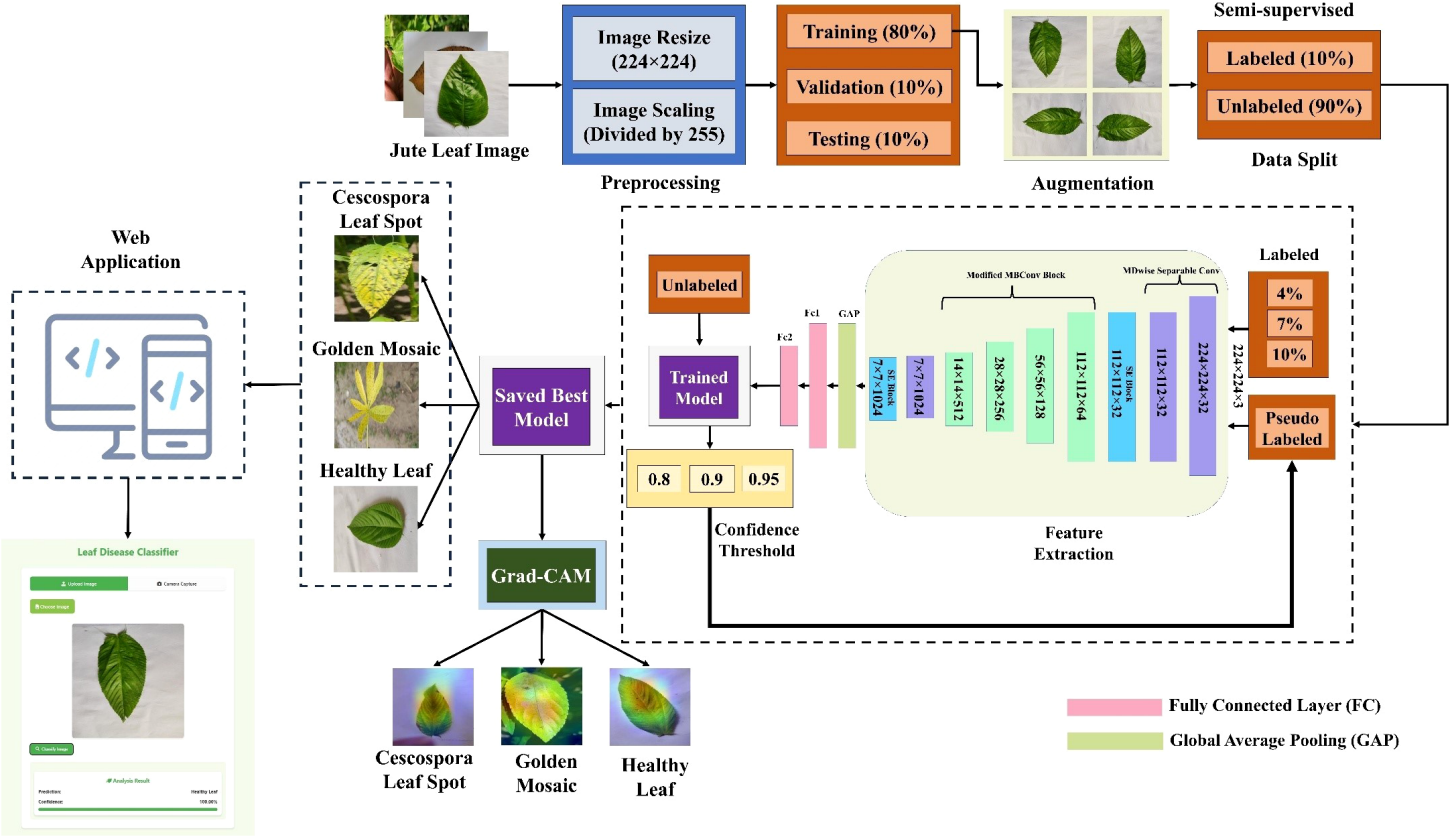
Workflow of the proposed semi-supervised self-training (ST) approach for jute leaf disease classification. The model is first trained on a small labeled subset (10%), predicts labels for unlabeled data (90%), selects high-confidence predictions as pseudo-labels, and iteratively retrains until convergence.

The whole classification process is divided into three parts. First, several well-established TL models were trained via supervised learning methods and evaluated across different performance metrics. Since the TL models did not perform as expected and had more complex architectures, a new lightweight custom CNN model was built using different components and compared with the TL models. **Figure 2** shows the workflow of jute leaf disease classification via the supervised method. After some trial and error, the final custom CNN model was found to outperform all the TL models. This new lightweight custom CNN model was subsequently utilized for the semi-supervised method. The model was initially trained on a small set of labeled data. Using this trained model, predictions were made on a large set of unlabeled data. The most confidently predicted images were treated as pseudo-labeled data. The model was then retrained on these pseudo-labeled images. This process was repeated several times until no pseudo-labeled images remained, ultimately resulting in the final, best-performing model. Finally, a web application was developed to facilitate real-time plant disease classification using the best-performing custom CNN model, thereby assisting farmers in accurate and timely diagnosis. **Figure 3** shows the proposed workflow for jute leaf disease classification using semi-supervised ST method. The feature extraction was done by the proposed model. Initially, the model used modified depthwise separable convolutions, which decomposed standard convolutions into depthwise and pointwise components, reducing computational cost while preserving spatial feature learning. To further enhance the sensitivity of the model to important features, SE blocks were integrated, which adaptively recalibrate channel-wise feature responses using global context. The modified MBconv blocks expanded the input features, applied grouped and depthwise convolutions, then compressed the features back while incorporating residual connections, enabling deeper and more efficient learning. Finally, the feature maps were aggregated using Global Average Pooling, producing a compact representation suitable for classification. This hierarchical structure allowed the model to progressively extract rich, discriminative features from raw input images.

### 3.4 Semi-supervised learning

The semi-supervised learning method handles partially labeled data to achieve higher classification levels (Wang et al., 2016). In semi-supervised learning, the collection of patterns is categorized into two subsets of D : 1) labeled data $\left\{D_{L}\}=\{(x_{i},y_{i})|i=1,\ldots ,l\right\}$ , where the pattern is denoted by x , y is the default label for x and the number of labeled instances is l ; and 2) unlabeled data $\left\{D_{U}\}=\{(x_{j})|j=l+1,\ldots ,l+u\right\}$ , where the pattern is denoted by x and the number of unlabeled instances is u . Usually, $∥D_{U}∥\gg ∥D_{L}∥$ . One advantage of semi-supervised learning is that it reduces the need for a large amount of labeled data, particularly in domains where the quantity of available labeled data is scarce. When no previously labeled datasets are available, it is common for an expert to manually classify the data in specific fields. The semi-supervised learning method handles partially labeled data to achieve higher classification levels. When an expert only recognizes a portion of the patterns in a given dataset, it becomes very challenging for them to categorize instances to increase the training set of data. This highlights yet another advantage of this type of learning (Chapelle et al., 2009). In the literature, ST is capable of handling semi-supervised datasets (Yarowsky, 1995).

#### 3.4.1 Self-training

Self-training is perhaps the earliest concept for categorizing unlabeled data from a lower percentage of previously classified data. The findings in the feature selection domain indicate that the wrapper algorithm uses a supervised approach to guide its decision-making process (Van Engelen and Hoos, 2020). ST is a wrapper algorithm that initiates training exclusively on labeled data and subsequently applies a supervised learning technique repeatedly. At each phase, the present decision function is employed to label a portion of the unlabeled instances. The supervised algorithm is once again retrained with its predictions using the additional labeled cases (Chapelle et al., 2009). A classifier is first developed that uses a limited amount of labeled data during the ST process. The classifier is subsequently utilized for the classification of unlabeled data. The training set comprises cases identified with the highest confidence index along with their expected labels. The classifier undergoes retraining until the unlabeled dataset is fully utilized, at which point the entire procedure is repeated. This method involves the classifier acquiring knowledge through its own predictions, hence the term ST (Zhu and Goldberg, 2009). A confidence parameter was introduced to the ST algorithm in (Rodrigues et al., 2013) as an extension, functioning as a threshold for incorporating new cases into the labeled dataset. New examples are incorporated into the labeled dataset when their prediction confidence meets or exceeds the threshold, defined as the minimum confidence rate (0.9) for the inclusion of new instances.

#### 3.4.2 Confidence regularization self-training

**Figure 4:**
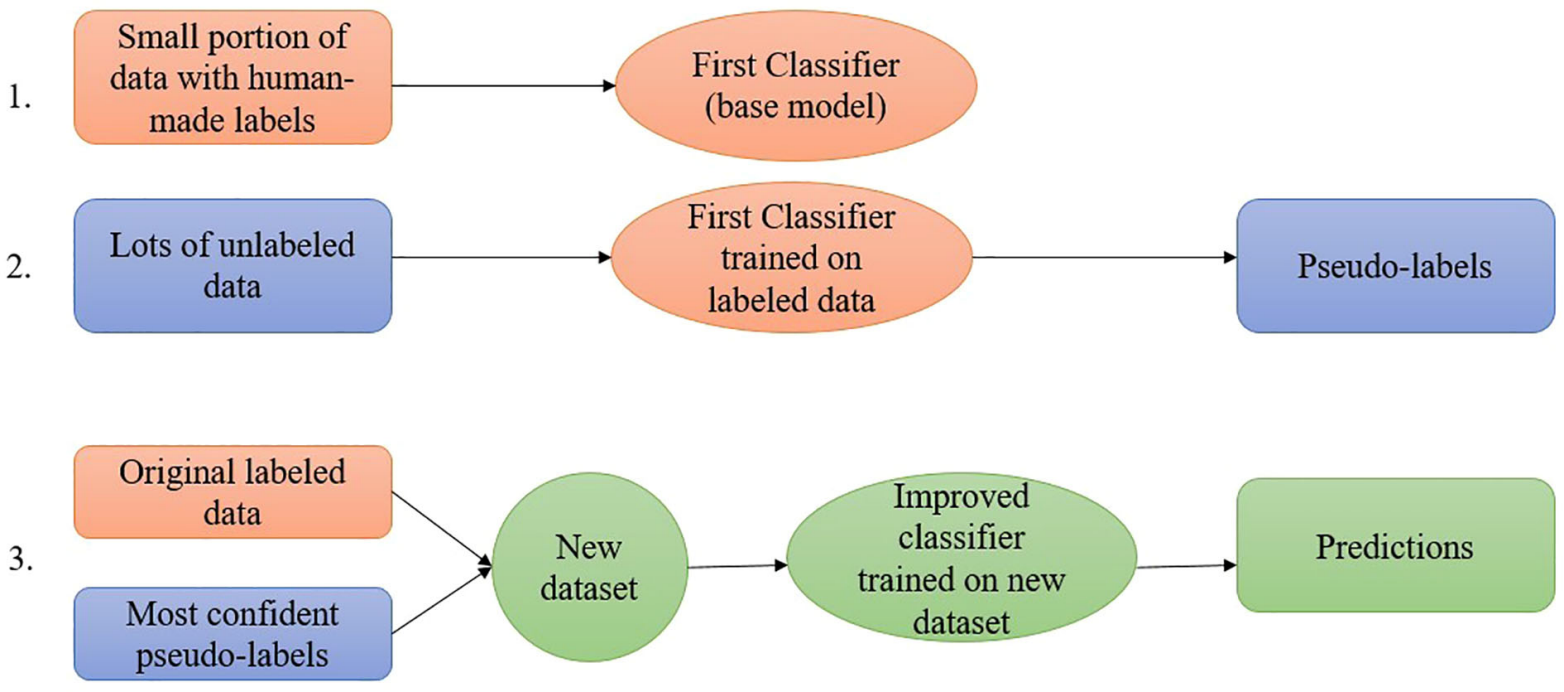
Block diagram of the confidence regularization self-training process. A supervised classifier trained on labeled data predicts unlabeled data, selects high-confidence data above a threshold, assigns pseudo-labels, and adds them to the labeled set in iterative cycles until all unlabeled data are processed.

**Figure 4** represents the step-by-step process of the confidence regularization ST process. A supervised classifier is initially developed using the labeled dataset in the ST process. This classifier is subsequently employed to classify the unlabeled data. A new confidence threshold value is calculated and applied to select additional cases for labeling. Cases with a prediction confidence value that meets or exceeds the confidence threshold are selected and labeled through a series of established procedures in the subsequent phase. The procedure continues until the unlabeled dataset is depleted, and the newly labeled dataset is utilized for ongoing operations. Algorithm 1 presents the sequential procedure of the newly developed ST version for this work.

Unlike the ST extension suggested in the literature (Rodrigues et al., 2013), the ST proposed in this work allows changing the stated value for each iteration, which does not change the confidence threshold to account for new instances (Algorithm 1, Line 4).

The label predicted by the classifier is assigned immediately to an unlabeled instance and then transferred to the pseudo-labeled dataset. Various approaches to choose the appropriate label are proposed in this study (Algorithm 1, Line 6).

Algorithm 1 **Self-Training with Confidence Adjusting.**


1: **Input:** labeled data 
$\left\{D_{L}\right\}$
, unlabeled data 
$\left\{D_{U}\right\}$
;
2: Initially, we have 
$\{D_{L}\}=\{(x_{i},y_{i})|i=1,\ldots ,l\}\;$
and 
$\left\{D_{U}\}=\{(x_{j})|j=l+1,\ldots ,l+u\right\}$
;
3: Train classifier 
C
 on 
$\left\{D_{L}\right\}$
;
4: Apply 
C
 on instances of 
$\left\{D_{U}\right\}$
;
5: Calculate a new value for confidence rate.
6: Remove a subset 
$S=\{s_{1},s_{2},\ldots ,s_{n}\}$
 from 
$\left\{D_{U}\right\}$
, so that the confidence rate in 
C(x)
 is greater than or equal to the minimum confidence rate for new instances to be included; Use different strategies to choose the label for every instance in subset 
S;

7: Add 
{(x, C(x))|x ∈S}
 to set 
$\left\{D_{L}\right\}$
 until 
$\{D_{U}\}=\emptyset$

8: **Output:** Labeled data

### 3.5 Proposed CNN architecture

#### 3.5.1 Modified depthwise separable convolution

In conventional depthwise separable convolution, a standard convolution is split into a depthwise convolution applying a single filter per input channel and a 1×1 pointwise convolution that combines these outputs, resulting in reduced computational cost and improved efficiency. However, the computational cost can be further reduced by using the principle of group convolutions, where the input and output channels are divided into separate groups and convolutions are performed independently within each group. This reduces the number of parameters and operations compared with standard convolutions while still maintaining representational power. In standard depthwise separable convolution, depthwise and pointwise operations are performed as shown in Equations 1 and 2:


(1) $$\text{Cos}t_{depthwise}=D_{k}\times D_{k}\times H\times W\times C_{in}$$

(2) $$\text{Cos}t_{po\mathrm{int}wise}=H\times W\times C_{in}\times C_{out}$$

where $D_{k}\times D_{k}$ represents the kernel dimension of the depthwise convolution, H×W×C denotes the input feature map, and $C_{in}$ and $C_{out}$ represent the total number of input and output channels, respectively. To reduce the computational complexity while preserving the information, the pointwise convolution is replaced with a grouped pointwise convolution with a G number of groups. Equation 3 shows the cost calculation of the grouped convolution.


(3) $$\text{Cos}t_{grouped}=\frac{H\times W\times C_{in}\times C_{out}}{G}$$

In this study, a group count of G=4 was chosen, leading to a significant reduction in the parameter count while still enabling information flow between the groups of channels. The output of the grouped pointwise convolution is normalized via group normalization (GN), which stabilizes the training process by normalizing the input within each group of channels, improving generalization, and making the model less sensitive to the batch size. Unlike Batch Normalization, which computes statistics across the batch dimension, GN divides channels into groups and computes normalization within each group, making normalization independent of the batch size. The mathematical expression for group normalization is shown in Equation 4.


(4) $$GN=\frac{x-\mu_{g}}{\sqrt{\sigma_{g}^{2}+\epsilon }}$$

where x is the input to the activation function and σ represents the sigmoid activation function. Here, $\mu_{g}$ and $\sigma_{g}^{2}$ are the mean and variance computed over all the elements in the group. ϵ is a small constant added to avoid division by zero. Finally, a Swish activation function is used to introduce smooth, non-linear transformations after the modified depthwise separable convolution, which enhances gradient flow and feature expressiveness. Equation 5 represents how the swish activation function works.


(5) swish=x·σ(x)

The following expression shown in Equation 6 summarizes the whole process of modified depthwise separable convolution:


(6) $$MDwiseSepConv_{k\times k}=swish(GN(Conv2D_{1\times 1}^{G}(DwiseConv_{k\times k}(X))))$$

where k×k is the dimension of the kernel and G is the number of groups for grouped convolution. DwiseConv denotes the depthwise convolution applied to the input tensor, and MDwiseSepConv is the output of the modified depthwise separable convolution.

**Figure 5:**
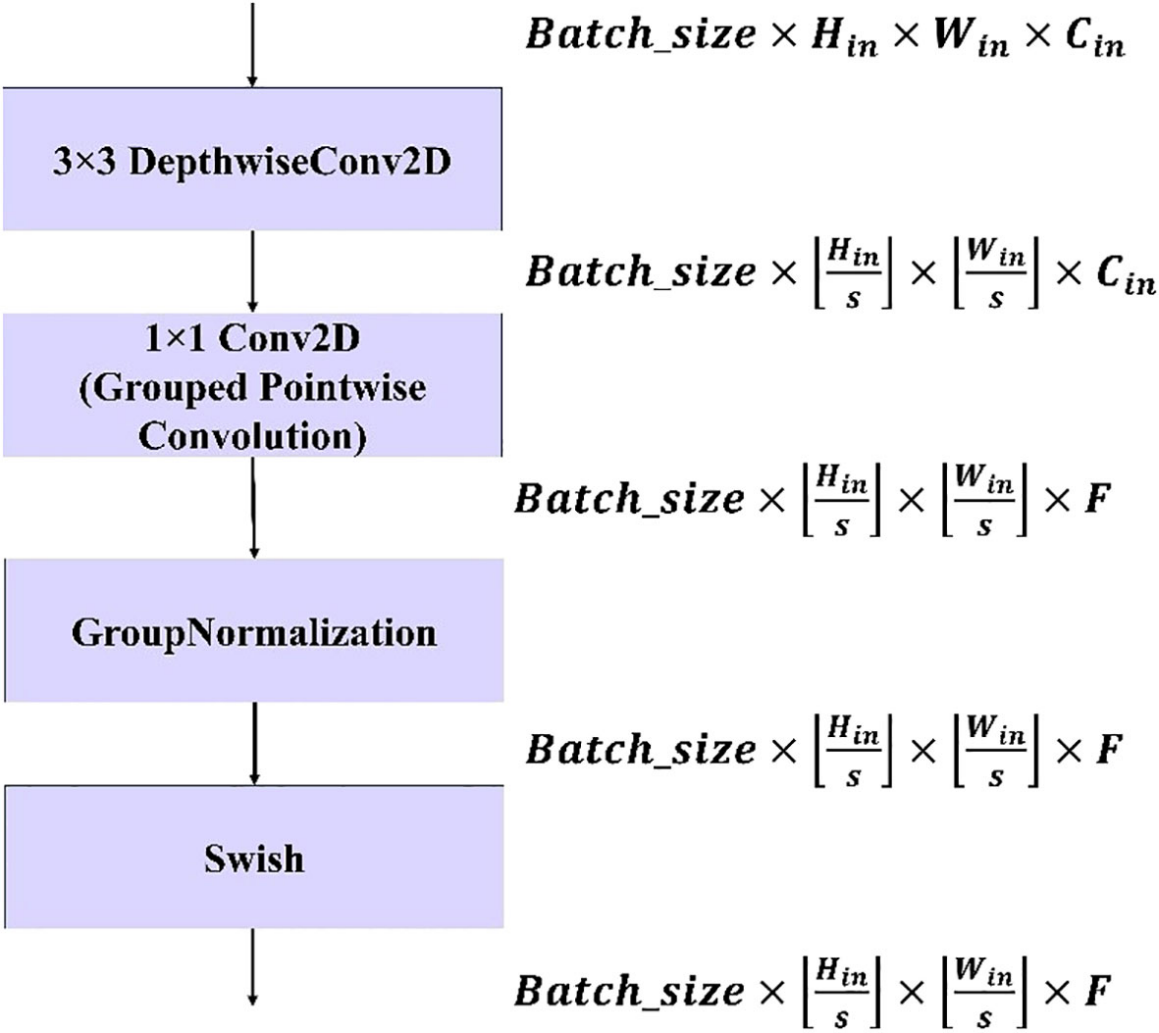
Workflow of the proposed modified depthwise separable convolution block. The block consists of a 3×3 depthwise convolution, a 1×1 grouped pointwise convolution, group normalization, and a Swish activation function. The tensor dimensions at each stage are indicated on the right, showing how input size $\left(H_{in},W_{in}\right)$ , stride ( s) , and filter count (F) affect the output feature maps.

The modified depthwise separable convolution block shown in **Figure 5** is used in this model to significantly reduce the computational cost while maintaining strong representational power. By replacing the standard pointwise convolution with a grouped pointwise convolution, the model benefits from a reduced parameter count and fewer operations. Additionally, the use of Group Normalization (GN) helps stabilize the training process by normalizing the activations within each group, making the model more robust and independent of the batch size. Unlike batch normalization, which depends on the entire batch, the GN operates on smaller groups of channels, improving generalizability. The final enhancement comes from the use of the Swish activation function, which introduces smooth, non-linear transformations, leading to better gradient flow and richer feature expressiveness. This combination of techniques optimizes the efficiency and performance of the model, making it particularly suitable for applications with limited computational resources, such as mobile and embedded systems.

#### 3.5.2 Modified squeeze and excite block

To enhance the squeeze and excite (SE) operation, GN was incorporated with the SE block. Using GN after the SE operation helps stabilize training and improve feature representation by normalizing channel-wise activations within each group. GN was chosen instead of Batch Normalization (BN) because it does not rely on batch statistics and therefore remains stable when training with small batch sizes, which were necessary in this study due to hardware limitations. By normalizing across groups of channels, GN reduces inter-channel variance and ensures consistent feature scaling. This, in turn, improves the effectiveness of the SE block in modeling long-channel dependencies. Specifically, GN ensures that the recalibration performed by SE focuses on meaningful inter-channel relationships rather than noise introduced by unstable normalization statistics (Wu and He, 2018). Instead of using the ReLU function in the excitation operation, Swish activation was employed, which provides smoother gradients and improved learning dynamics, ultimately enhancing the model’s performance. These modifications within the SE block improve the conventional SE module with Batch Normalization and the ReLU activation function. The modified SE block operation can be described as follows:

The squeeze operation using global average pooling converts each channel to a single numerical value as shown in Equation 7:


(7) y=GlobalAveragePooling2D(x)

Then, the squeezed vector is passed through a fully connected layer followed by the swish activation function. The activated output was passed through another dense layer to restore the original number of channels, followed by GN and the sigmoid activation function as shown in Equation 8:


(8) $$z=\sigma (GN(W_{2}(swish(W_{1}(y)))))$$

where $W_{1}$ and $W_{2}$ are the weights of the fully connected layer. The attention weights are reshaped to match the input tensor’s channel dimensions and broadcast across spatial dimensions. Finally, these weights are multiplied element-wise with the input tensor to recalibrate the feature maps as performed in Equation 9.


(9) SE=x⊙reshape(z)

Where, the element-wise multiplication is denoted by ⊙ and where SE is the final output of the SE block.

**Figure 6:**
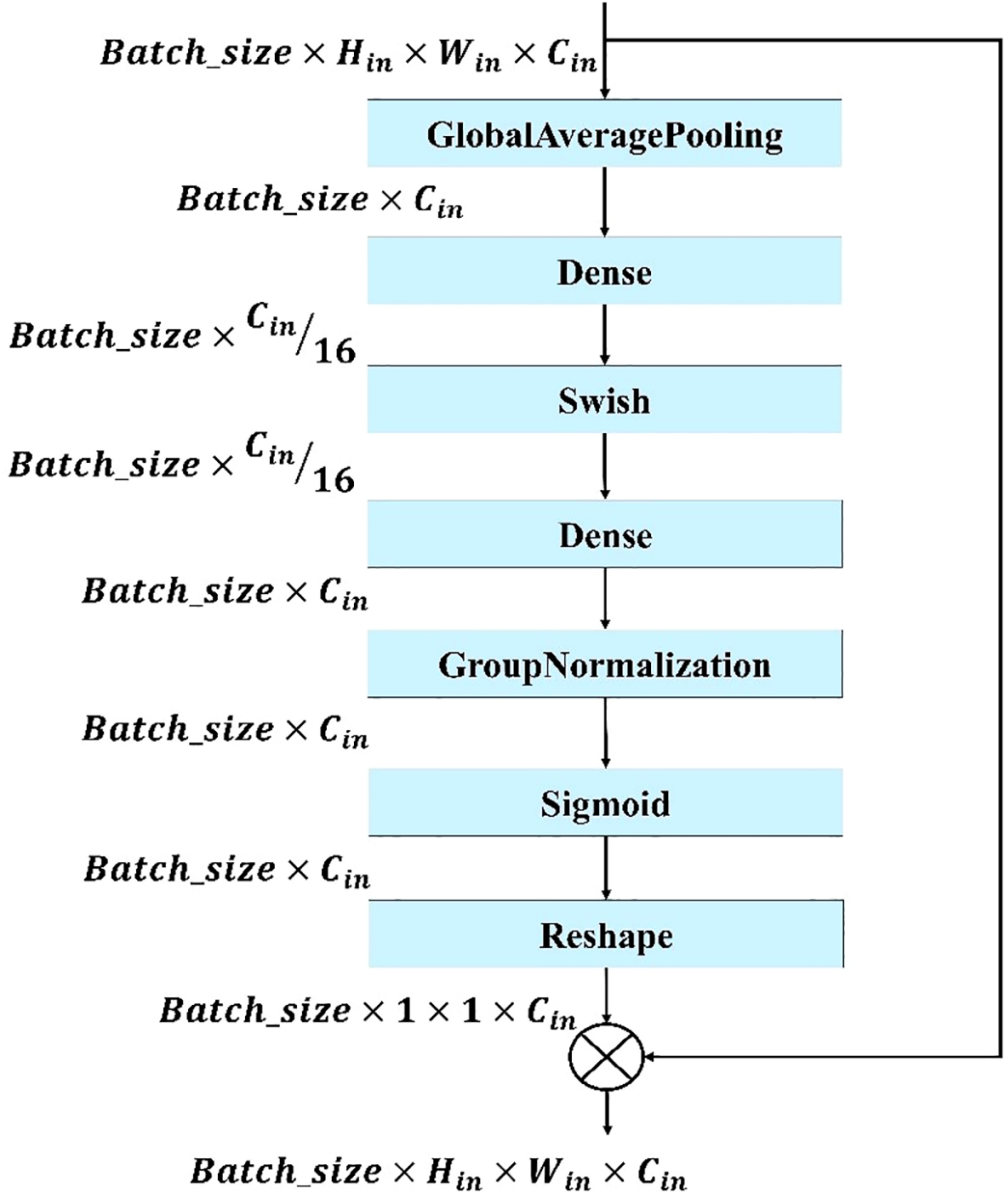
Structure of the proposed modified SE block. The block applies global average pooling, two dense layers with Swish activation, group normalization, and a sigmoid function to generate channel-wise attention weights, which are reshaped and multiplied with the input feature maps to recalibrate channel importance.

The modified SE block shown in **Figure 6** is used in this model to enhance feature recalibration and improve the model’s ability to focus on the most informative features. It improves the traditional SE module by providing more efficient and accurate channel-wise attention, helping the model to better identify and emphasize important features while suppressing less relevant ones. This results in improved feature representation, more effective training, and higher performance across a range of tasks.

#### 3.5.3 Modified mobile inverted bottleneck block

The proposed modified mobile inverted bottleneck convolution (MBconv) block is inspired by the MobileNetV2 architecture (Sandler et al., 2018). Several modifications have been made to make the model more lightweight and efficient. Several modifications were introduced to improve both the efficiency and representational power while keeping the computational cost low. Like MobileNetV2, the block starts with an expansion phase as shown in Equation 10, where the input tensor $X\in {\mathbb{R}}^{H\times W\times C_{in}}$ is projected to a higher-dimensional space, expanding the channel dimension by a factor of r , resulting in an intermediate tensor $X_{1}\in {\mathbb{R}}^{H\times W\times (r\cdot C_{in})}$ .


(10) $$X_{1}=swish(Conv_{1\times 1}(X))$$

This is followed by depthwise convolution as shown in Equation 11, which efficiently captures spatial relationships with minimal parameter cost:


(11) $$X_{2}=swish(DwiseConv_{3\times 3}(X_{1}))$$

Unlike the original MBConv, the projection phase in this modified block uses a grouped pointwise convolution instead of a standard 1×1 convolution. This operation reduces the number of output channels while also enforcing sparsity and group-wise feature learning. To further enhance channel interdependencies, the SE block is applied to adaptively recalibrate the feature maps as the following Equation 12:


(12) $$X_{3}=SE(GN(Conv_{1\times 1}^{G}(X_{2})))$$

A residual connection is formed by applying the modified depthwise separable convolution to the skip path as shown in Equation 13, ensuring consistent feature alignment and dimensionality regardless of input–output shape compatibility. This replaces the conditional identity mapping used in traditional residual blocks:


(13) $$R=MDwiseSepConv_{1\times 1}(X)$$

This ensures that the skip path always undergoes a learnable transformation, promoting better feature fusion and flexibility across varying spatial or channel configurations. Finally, the residual and transformed features are fused via element-wise addition and passed through a non-linear activation as expressed in Equation 14:


(14) $$Y=swish(X_{3}⊕R)$$

where ⊕ denotes element-wise addition. This modified MBConv block combines the depthwise efficiency of MobileNetV2 with SE attention mechanisms and grouped convolutional compression, resulting in a lightweight yet expressive building block for modern convolutional neural networks.

**Figure 7:**
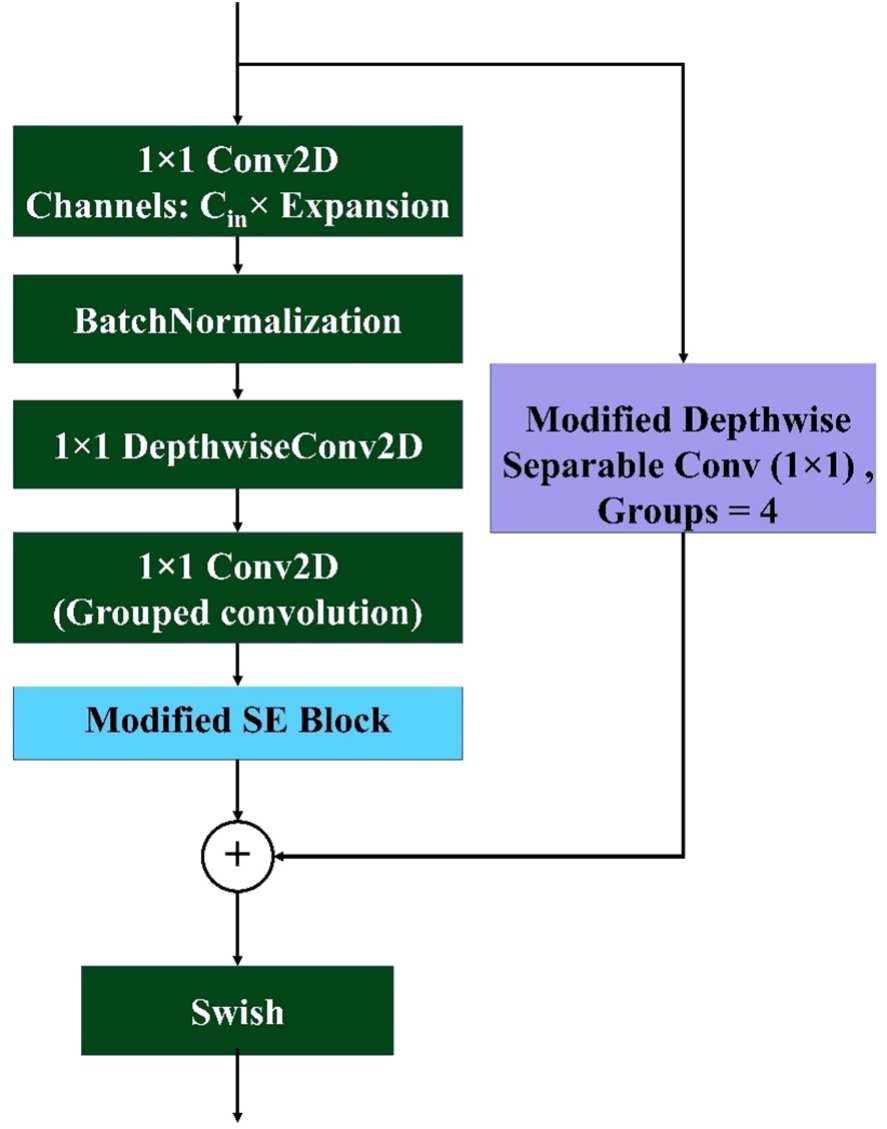
Architecture of the proposed modified MBconv block. The block includes an expansion phase with 1×1 convolution, depthwise convolution, grouped pointwise convolution, and a modified SE block. A skip connection with a modified depthwise separable convolution ensures feature alignment, followed by element-wise addition and Swish activation for non-linearity.

**Figure 8:**
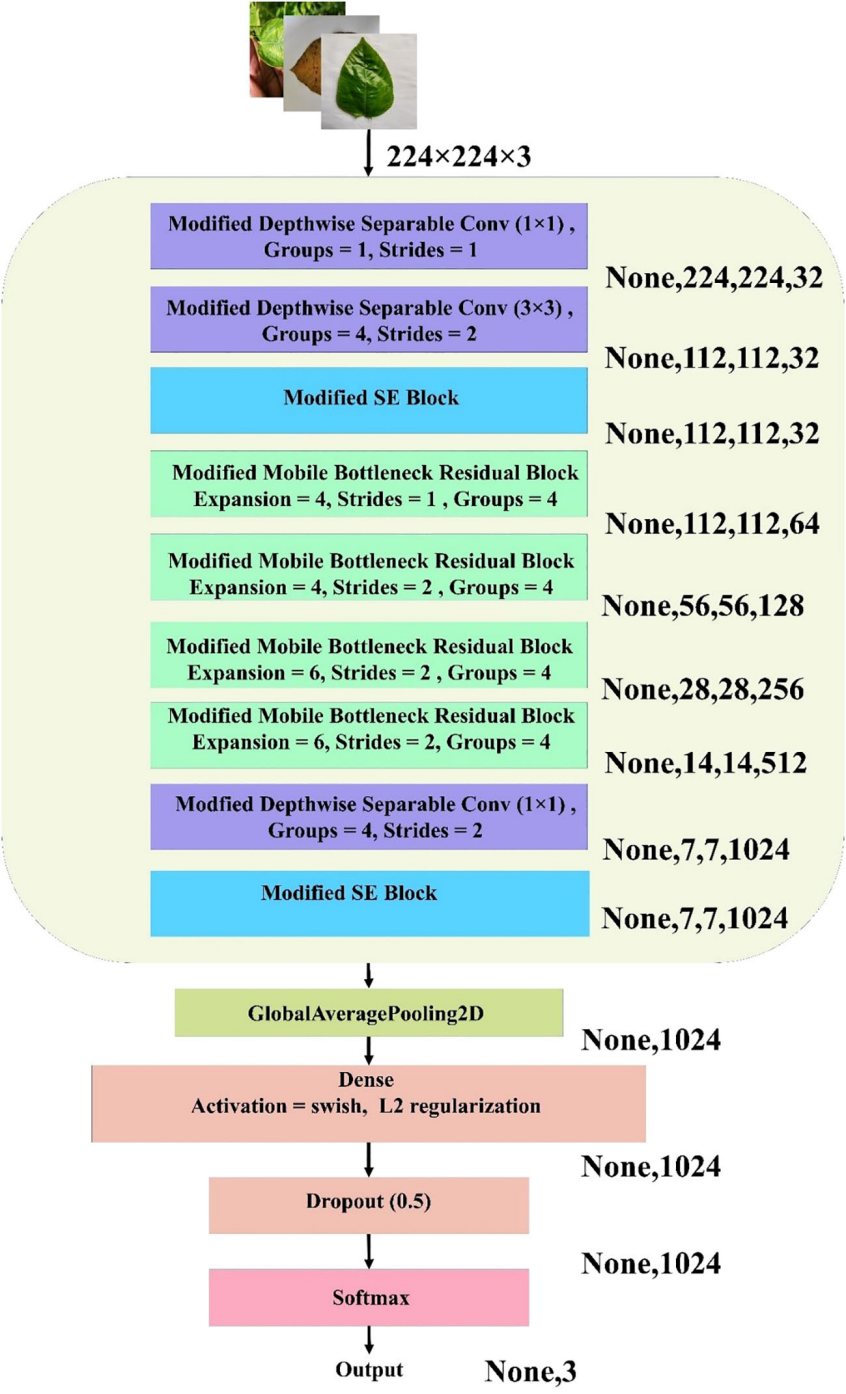
Architecture of the proposed lightweight CNN for jute leaf disease classification. The model combines modified depthwise separable convolutions, SE blocks, and MBconv blocks with grouped convolutions. Global average pooling, dense layers with Swish activation and L2 regularization, dropout, and a softmax layer produce the final three-class output. Output dimensions at each stage are shown on the right.

The MBConv Block shown in **Figure 7** is utilized in this model to achieve a balance between computational efficiency and representational power. By leveraging depthwise convolution and grouped pointwise convolution, this block significantly reduces the number of parameters and operations, making it lightweight and well suited for resource-constrained environments. The inclusion of the Squeeze and Excite (SE) block enhances the channel-wise attention mechanism, allowing the model to adaptively recalibrate feature maps and emphasize important information. The residual connection with modified depthwise separable convolution ensures that feature alignment and dimensionality are maintained, even if the input–output shape differs, while promoting better feature fusion. Overall, this modified MBConv block provides a highly efficient and expressive convolutional building block that can improve the model’s accuracy and robustness without incurring a significant computational cost, making it ideal for lightweight yet powerful neural network architectures. **Figure 8** represents the overall architecture, and the modifications made to the proposed custom CNN model.

### 3.6 Explainable AI

A DL visualization technique called Grad-CAM is used to comprehend model choices, particularly in computer vision tasks. The method creates a heatmap by using the target class’s gradients in relation to the last convolutional layer. Class-specific details in the input image can be visualized because the final convolutional layers balance spatial information and high-level semantics (Saranya and Subhashini, 2023).

Grad-CAM makes use of the rich information in the final layer of data by highlighting areas where the model focuses its attention to produce unique patterns. The algorithm effectively highlights important regions in the input image that contribute to target class prediction by computing gradients of the class score with respect to feature maps, performing weighted combinations, and producing a heatmap (Islam et al., 2022). Equation 15 summarizes the operation done by Grad-CAM.


(15) $$L_{c}^{CAM}=\sum_{i}\sum_{j}w_{c}^{k}A_{k}^{ij}$$

where $L_{c}^{CAM}$ is the localization map for class c in Grad-CAM. The association with the k -th feature map for class c is denoted by $w_{c}^{k}$ . $A_{k}^{ij}$ is the activation for the k -th feature map at a spatial location (i, j). This equation represents the formulation of Grad-CAM for generating a class-specific localization map through the combination of feature weights $w_{c}^{k}$ and activation $A_{k}^{ij}$ from different spatial locations. After generating the heatmaps with the help of Grad-CAM, the regions were marked with red bounding boxes by mapping the color from the heatmap to indicate the location of diseased area.

### 3.7 Web application

A functional Android app prototype was developed to demonstrate the real-world application and capabilities of the established model using Flask-based web application. Flask is a lightweight and flexible web application framework in Python that simplifies the deployment of ML, DL and computer vision models as interactive web applications. It integrates seamlessly with popular Python libraries commonly used in these workflows, including those for data processing, model inference, and visualization. This compatibility enables developers to build responsive and user-friendly interfaces for showcasing their DL models and results. Flask’s minimalistic design and modular architecture also allow for greater customization and control, making it a suitable choice for developing tailored applications in real-world scenarios. The CNN model, after training, was saved in a standard.h5 format and loaded into the Flask backend for inference. Upon receiving an image from the user, the server processed the input by resizing it to 224 × 224 pixels and normalizing the pixel values to match the model’s input requirements. The preprocessed image was then passed through the CNN model to perform inference. The Flask application returned the classification results identifying whether the leaf is healthy or affected by any disease classes.

### 3.8 Hyperparameter and system configuration

To achieve a high level of accuracy with the suggested model, it is necessary to carefully adjust and fine-tune its hyperparameters. The values of these hyperparameters are presented in **Table 3**. Empirical evidence suggests that the Adamax optimizer outperforms other widely used optimizers in terms of achieving higher rates of learning. In addition, batch normalization layers were included to expedite the training of the model.

**Table 3:** Hyperparameter configurations used in this work.

Hyperparameters	Value
Batch size	16
Optimizer	Adamax
Initial learning rate	0.001
Epochs	50
LR reduce factor	0.1
Loss function	Sparse Categorical Crossentropy
Kernel regularizer	L2=0.01

A balance between memory consumption and training speed influences practical decisions regarding batch size. In this research, a batch size of 16 was employed for normalization. Following numerous experiments conducted with training data from the combined dataset, the remaining hyperparameter values were selected somewhat arbitrarily. A trial-and-error methodology was employed to iteratively fine-tune the hyperparameters for optimal performance. **Table 4** represents the system configuration used in this work.

**Table 4:** System configuration used in this work.

Tools	Configuration
Programming Language	Python
Backend	Keras with TensorFlow
GPU RAM	15 GB
Disk Space	78.2 GB
System RAM	12.72 GB
GPU	Nvidia Tesla T4
Operating system	windows 10
Input	Jute Leaf
Input Size	224×224

### 3.9 Performance metrics

Several well-known evaluation metrics were computed to assess the classification performance of the proposed CNN model. To evaluate the model’s performance, a confusion matrix was generated based on the model’s prediction on the test data. Evaluation metrics such as accuracy, precision, recall, F1 score and AUC were computed to differentiate between different models (Hasan et al., 2024). The following metrics were derived via the established methodologies. Equations 16–19 represent the mathematical expressions for the metrics used to evaluate the proposed model.


(16) $$Accuracy=\frac{TP+TN}{TP+TN+FP+FN}$$

(17) $$Recall=\frac{TP}{TP+FN}$$

(18) $$Precision=\frac{TP}{TP+FP}$$

(19) $$F1-score=\frac{2\times precision\times recall}{precision+recall}$$

Here, FP = false positive, TP = true positive, FN = false negative, and TN = true negative. TP indicates when the model correctly identifies a diseased leaf as diseased. FP indicates when the model incorrectly predicts a healthy leaf as diseased. FN indicates when the model incorrectly predicts a diseased leaf as healthy. TN indicates when the model correctly identifies a healthy leaf as healthy.

## 4 Results

The following sections present a thorough analysis of the experimental results, examining the performance of the proposed methodology in comparison to established TL benchmarks. A detailed discussion has been presented to contextualize these findings, interpret their implications, and highlight the significance of the research outcomes in the broader context of jute leaf disease detection.

### 4.1 Result analysis of the supervised and semi supervised self-training method

A lightweight custom CNN model was developed for deployment in both supervised and semi-supervised learning frameworks, incorporating three key architectural enhancements: Modified Depthwise Separable Convolution, Modified SE Block, and Modified MBConv. **Table 5** presents a comprehensive evaluation of the model’s performance across various configurations, each designed to isolate the impact of individual and combined modifications. Compared with conventional convolution, the CNN network utilizing only modified depthwise separable convolution (Custom 1) achieved an accuracy of 92.63% while significantly reducing the number of parameters. It further reduced the number of parameters by incorporating a grouped convolution mechanism within the pointwise convolution, enhancing computational efficiency. This resulted in less computation and more efficient feature extraction without compromising performance. After using the modified SE block with the Custom 1 network (Custom 2), the results drastically improved from 92.63% to 96.84%accuracy, indicating that the dynamic channel-wise recalibration significantly enhanced feature representation and overall model performance. This highlighted the critical need for an attention mechanism to guide the network in focusing on the most informative features, thereby improving learning efficiency and overall performance. The modified MBConv blocks were subsequently incorporated without the SE block and without the modified depthwise separable convolution in the skip connections to build the Custom 3 network, which achieved an accuracy of 95.79%. Although this accuracy was slightly lower than the 96.84% accuracy of Custom 2, it significantly outperformed Custom 1, demonstrating the strength of the mobile inverted bottleneck block in enhancing feature extraction. Compared with a network relying solely on modified depthwise separable convolutions, the use of MBConv blocks provided deeper feature representation and better information flow through efficient expansion, depthwise filtering, and compression mechanisms, leading to substantially improved learning capacity. Integrating the modified SE block within the modified MBConv structure (Custom 4) further improved the accuracy to 97.89%, highlighting the effectiveness of the attention mechanism in enhancing feature extraction and boosting overall model performance. Finally, the proposed model, which combined all three blocks in the suggested configuration, achieved the highest accuracy of 98.95%, outperforming all the custom networks.

**Table 5:** Performance metric evaluation with different modifications of the proposed custom CNN model using supervised method.

Models	Customization elements			Performance metrics			
	Modified depthwise separable convolution	Modified SE block	Modified MBConv	Accuracy (%)	Precision (%)	Recall (%)	F-1 Score (%)
Custom 1	✔	✖	✖	92.63	93.30	92.67	92.91
Custom 2	✔	✔	✖	96.84	97.14	97.22	97.06
Custom 3	✖	✖	✔	95.79	95.81	96.18	95.95
Custom 4	✖	✔	✔	97.89	98.03	98.03	98.03
Proposed	✔	✔	✔	**98.95**	**98.99**	**99.07**	**99.02**

Bold values indicate best results.

The results indicated that while each architectural enhancement independently contributed to improved model performance, the integration of all three yielded the best outcomes. Specifically, the proposed model achieved the highest scores across all the performance metrics, with an accuracy, precision, recall, and F1 score of 98.95%, 98.99%, 99.07% and 99.02% respectively, underscoring the effectiveness of the combined architectural improvements. This demonstrated the potential of the proposed custom CNN design in achieving high performance while maintaining computational efficiency.

To evaluate the effect of key design choices, an ablation study was conducted by varying the group size, expansion ratio, and activation function under the supervised learning method. After achieving the best results from the final model architecture through ablation study in the supervised learning was used for the semi-supervised method without conducting any further ablation study. The effect of grouped convolutions with various group sizes were analyzed. The effect of expansion ratio of each modified MBConv block and how the choice of activation function impacted the results were also examined. From **Table 6**, it can be seen that when ReLU was used as the activation function with group size = 4 and expansions = (4, 4, 4, 4), the model achieved 93.33% accuracy, 93.99% precision, 93.99% recall, and 93.75% F1-score, indicating relatively weaker performance. Replacing ReLU with Swish activation under the same configuration significantly improved the results to 98.89% accuracy, 99.02% precision, 98.92% recall, and 98.96% F1-score, showing that Swish activation enhances gradient flow and representational learning. Increasing the expansions uniformly to (6, 6, 6, 6) while keeping Swish activation reduced performance to 95.56% accuracy, 95.83% precision, 95.95% recall, and 95.83% F1-score, suggesting that overly high expansion introduced unnecessary complexity. A mixed expansions of (4, 4, 6, 6) with Swish achieved the best performance, reaching 98.95% accuracy, 98.99% precision, 99.07% recall, and 99.02% F1-score, confirming the effectiveness of balanced expansion. Finally, increasing the group size to 8 or 16 with the (4, 4, 6, 6) expansion and Swish activation led to performance drops (96.67% and 95.56% accuracy, respectively), showing that larger group sizes reduce inter-channel feature interaction and limit learning capacity. These results demonstrated that the optimal combination was group size = 4, expansions = (4, 4, 6, 6), and Swish activation, which maximized accuracy and generalization while keeping the model lightweight.

**Table 6:** Performance comparison of the proposed CNN model under different group sizes, expansion ratios, and activation functions using the supervised learning method.

Group Size	Expansion s in four layers of Modified MBConv Block	Activation function	Accuracy (%)	Precision (%)	Recall (%)	F-1 Score (%)
4	4, 4, 4, 4	ReLU	93.33	93.99	93.99	93.75
4	4, 4, 4, 4	Swish	98.89	99.02	98.92	98.96
4	6, 6, 6, 6	Swish	95.56	95.83	95.95	95.83
4	**4, 4, 6, 6**	**Swish**	**98.95**	**98.99**	**99.07**	**99.02**
8	4, 4, 6, 6	Swish	96.67	96.97	97.06	96.87
16	4, 4, 6, 6	Swish	95.56	96.08	96.08	95.83

The results show the impact of architectural choices on accuracy, precision, recall, and F1-score.

Bold values indicate best results.

The results reported in the **Tables 5** and **6** represented the best performance of the proposed model. However, to further verify the stability and reliability of the model, multiple independent runs were conducted under the same experimental setup. The average results across these runs, along with their standard deviations, are presented in **Table 7**. The model consistently achieved strong performance, with accuracy ranging from 96.84% to 98.95% with a mean accuracy of 98.32%. Similar consistency was observed in precision (mean 98.41%), recall (mean 98.47%), and F1-score (mean 98.43%). The relatively low standard deviations (≤0.84%) indicated that the model’s performance was stable and not highly sensitive to random initialization or variations across runs. These results reinforced the robustness and generalization ability of the proposed lightweight CNN, supporting its suitability for practical deployment in real-world agricultural settings.

**Table 7:** Multiple independent runs by the proposed custom CNN model using the supervised method.

Multiple runs	Accuracy (%)	Precision (%)	Recall (%)	F-1 Score (%)
Run - 1	97.89	98.03	98.03	98.03
Run - 2	98.95	98.99	99.07	99.02
Run - 3	98.95	98.99	99.07	99.02
Run - 4	96.84	97.03	97.11	97.05
Run - 5	98.95	98.99	99.07	99.02
Mean	98.32	98.41	98.47	98.43
Standard Deviation	0.84	0.78	0.79	0.78

To evaluate the effectiveness of the proposed model in scenarios with limited labeled data, which is a common challenge in agricultural settings, a confidence-based semi-supervised ST approach was implemented. This method involved varying the proportion of labeled data and adjusting the confidence threshold for pseudo-label selection, as summarized in **Table 8**. The results align with the overarching aim of the study, demonstrating the model’s strong generalization capabilities even when trained on minimal labeled data. At just 4% labeled data, the model achieved a commendable accuracy of 93.68% at a 0.95 confidence threshold. As the labeled portion increased to 7%, the performance improved significantly, with the accuracy increasing to 95.79% and corresponding gains in precision, recall, and the F1 score. When 10% labeled data were combined with 90% unlabeled data, the model attained its highest performance under the semi-supervised framework, achieving 97.89% accuracy along with equally strong values across all other metrics. These findings confirm the practicality and effectiveness of the proposed semi-supervised strategy, reinforcing its relevance for real-world agricultural applications where labeled data are often scarce and validating the broader goal of achieving high performance with minimal manual annotation.

**Table 8:** Performance metric comparison of the proposed model using semi-supervised learning method with different confidence thresholds and labeled data.

Labeled data proportion (%)	Confidence threshold	Accuracy (%)	Precision (%)	Recall (%)	F-1 Score (%)
4	0.8	91.58	94.74	92.75	92.15
4	0.9	92.63	92.48	93.40	93.14
4	0.95	93.68	94.74	94.44	94.11
7	0.8	94.74	95.25	94.86	94.96
7	0.9	94.74	95.50	95.37	95.10
7	0.95	95.79	96.30	96.30	96.08
10	0.8	96.84	97.03	97.11	97.05
10	0.9	96.84	97.14	97.22	97.06
10	0.95	97.89	97.80	98.15	97.93

**Figure 9:**
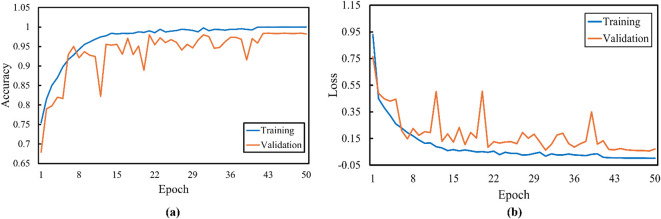
Proposed model **(a)** training accuracy and **(b)** loss curves for the traditional supervised learning method.

**Figure 9** shows that the proposed model achieves high training and validation accuracy. Although the training and validation curves showed minor fluctuations, they ultimately start plateauing after approximately 40 epochs. The loss curves demonstrate a rapid decrease in both training and validation loss in the initial epochs, followed by a more gradual decline. This finding indicated that the model learns effectively and generalizes well to the unseen data.

**Figure 10:**
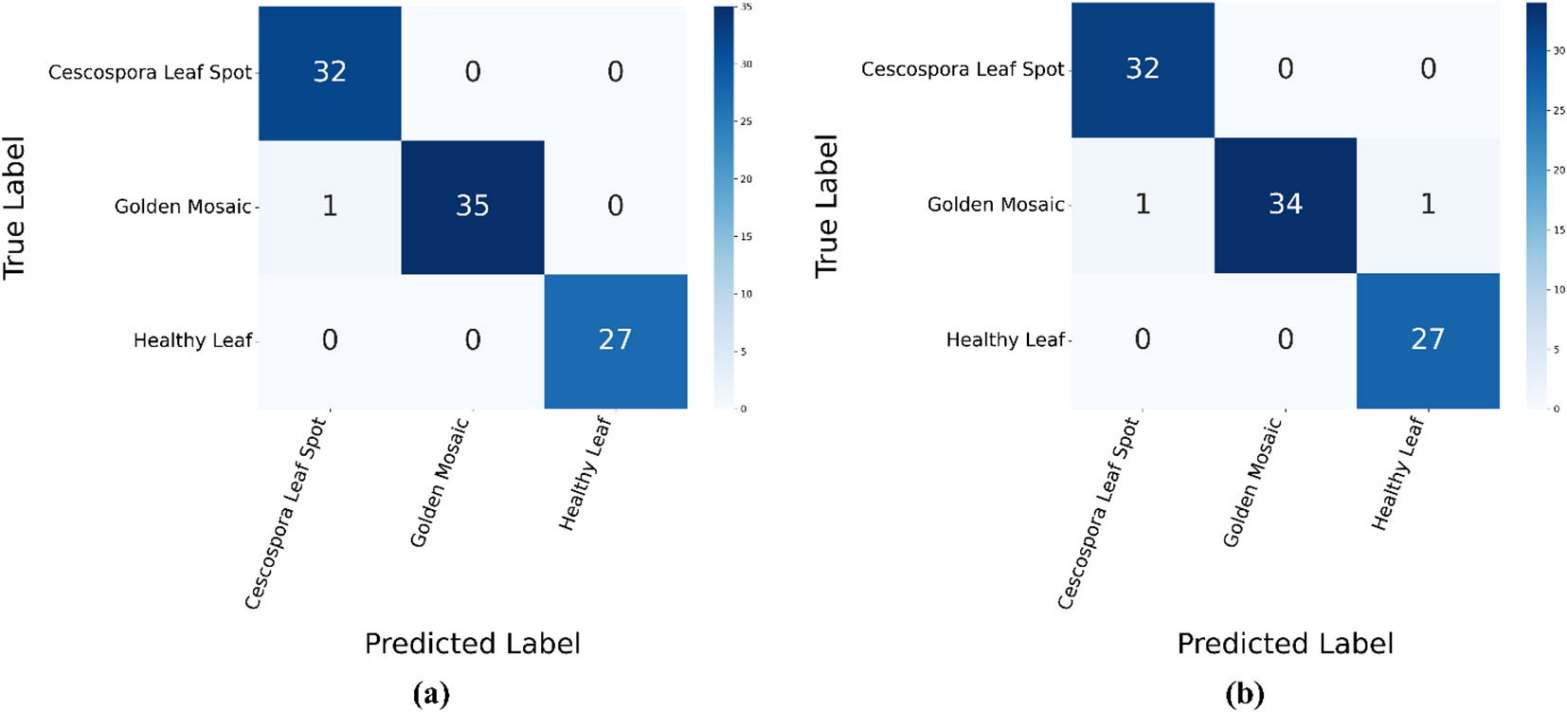
Confusion matrices for the proposed model using: **(a)** supervised method and **(b)** semi-supervised ST method.

**Figure 10** shows the confusion matrices for the proposed model using the traditional supervised method and semi-supervised ST method. In the supervised method, the model demonstrated high accuracy across all three classes: Cescospora Leaf Spot, Golden Mosaic, and Healthy Leaf, with minimal confusion between them. The semi-supervised ST method also showed strong performance, although there was a slight increase in confusion, particularly in Golden Mosaic. Overall, both methods achieved good classification accuracy, with the supervised method performing slightly better.

**Figure 11:**
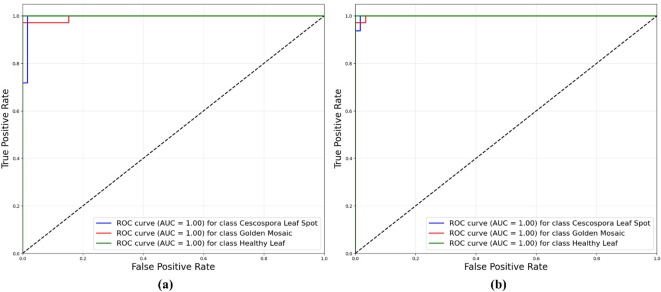
ROC curves for proposed model using: **(a)** traditional supervised method and semi-supervised ST method.

**Figure 11** illustrates the Receiver Operating Characteristic (ROC) curves for the proposed model, which compared the performances of the traditional supervised method and the semi-supervised ST method. The ROC curves plotted the true positive rate against the false positive rate for different classification thresholds. In the supervised method, the ROC curves for all three classes (Cescospora Leaf Spot, Golden Mosaic, and Healthy Leaf) reached an area under the curve (AUC) of 1.00, indicating perfect classification. The semi-supervised ST method also demonstrated strong performance, with AUC values of 1.00 for all three leaf classes. This showed that both methods were highly effective in distinguishing between different classes, with the traditional supervised approach exhibiting slightly superior performance.

### 4.2 Comparison with TLM

**Table 9** presents a comparative performance analysis of several TL models, including ResNet50, MobileNetV2, VGG19, Xception, and EfficientNetB7, which were evaluated using a supervised learning approach. Each model was selected based on its architectural strengths and relevance to the task. Among the evaluated models, MobileNetV2 demonstrated the highest classification accuracy, achieving 94.11%, thereby emerging as the top-performing model. Xception and EfficientNetB7 closely followed, attaining an accuracy of 93.68%. The performance of ResNet50 and VGG19 were slightly lower, with accuracies of 87.37% and 85.26%, respectively.

**Table 9:** Performance metric evaluation of the TL models using supervised method.

TL models	Accuracy	Precision	Recall	F-1 Score
ResNet50	87.37	89.01	87.85	87.98
MobileNetV2	94.11	94.72	95.14	94.78
VGG19	85.26	86.27	85.58	85.68
Xception	93.68	93.96	94.33	93.97
EfficientNetB7	93.68	93.60	94.33	93.77

### 4.3 Complexity analysis of the proposed model

Conducting a complexity analysis of the lightweight custom CNN model was crucial for assessing its efficiency in terms of training time, computational requirements, and model size, especially in comparison to conventional transfer learning architectures. As shown in **Table 10**, the proposed lightweight CNN model results in a markedly reduced number of trainable parameters (2.24 million) and a compact model size of 8.54 MB, which were significantly smaller than those of widely used models such as ResNet50 and EfficientNetB7. These characteristics highlighted the model’s computational efficiency, offering a lightweight solution without compromising classification performance. The reduced model complexity translated to faster training and easier deployment, making it well suited for real-time processing and deployment in resource-limited environments.

**Table 10:** Comparison of the parameter count and size of different models.

Model	Trainable parameter (million)	Total parameter (million)	Size (MB)
ResNet50	25.64	25.69	98.0
MobileNetV2	3.54	3.57	13.63
VGG19	20.55	20.55	78.40
Xception	22.91	22.96	87.60
EfficientNetB7	66.41	66.72	254.53
Proposed	**2.23**	**2.24**	**8.54**

Bold values indicate best results.

### 4.4 Grad-CAM visualization

**Figure 12:**
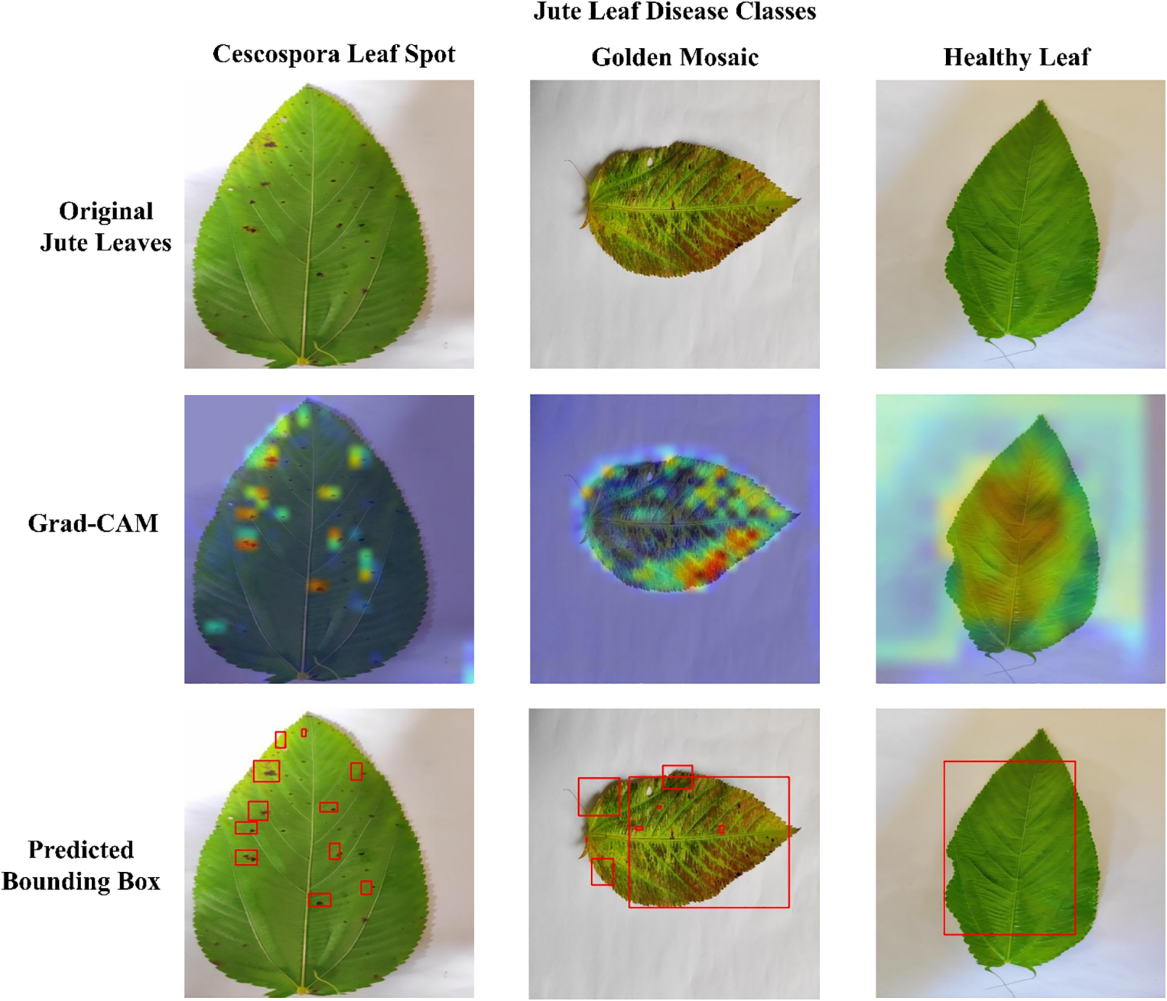
Grad-CAM visualizations demonstrating model explainability for each jute leaf class are shown. The first row contains the original jute leaf images of each class. The second row shows the corresponding Grad-CAM heatmaps, with warmer colors (red and yellow) indicating regions of greater model focus. The third row indicates the diseased area with red bounding boxes drawn from the Grad-CAM heatmaps.

**Figure 13:**
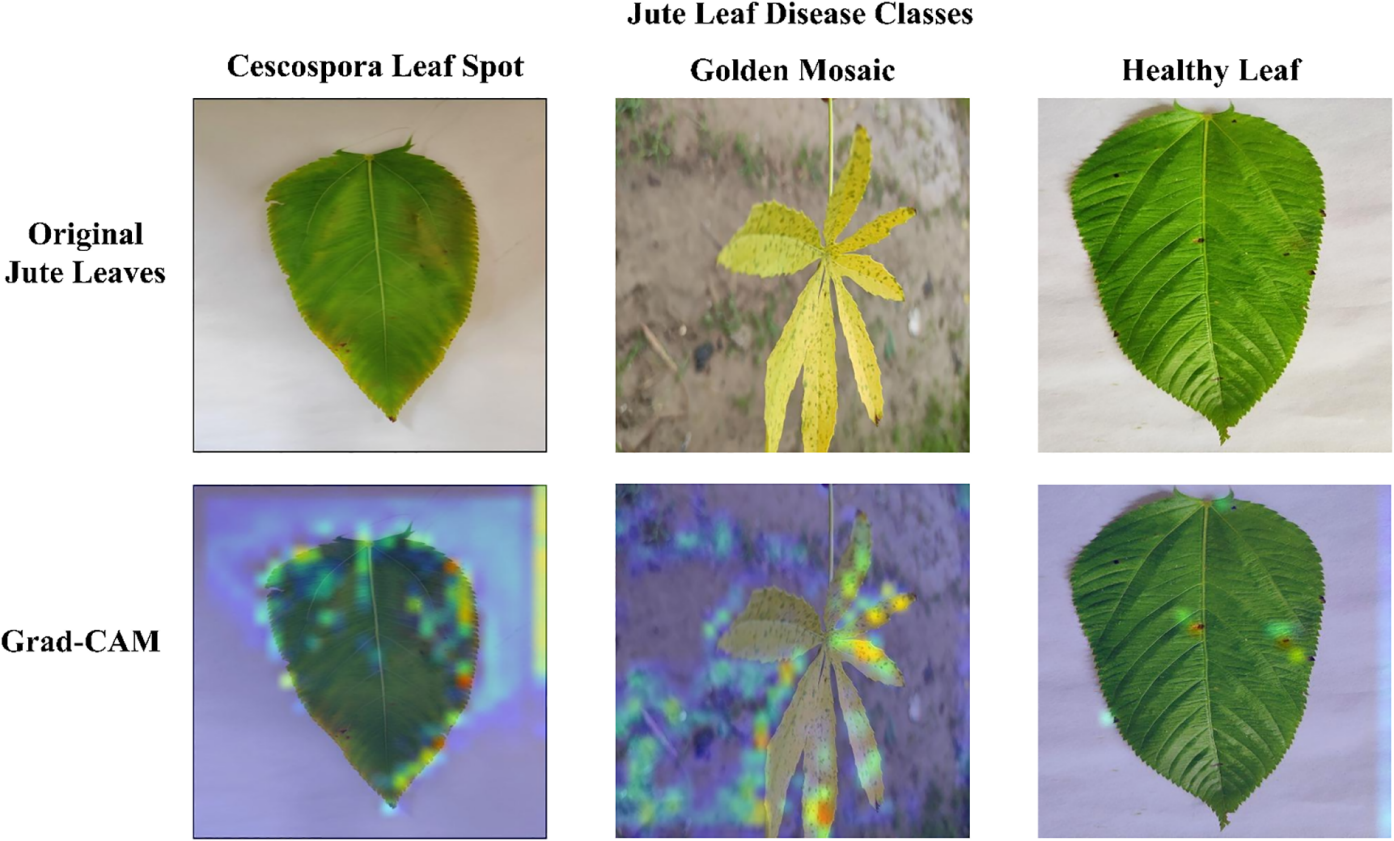
Grad-CAM visualizations for misclassified images showing that the model’s attention focused on irrelevant or misleading regions, contributing to incorrect predictions, without generating any bonding boxes.

**Figure 12** presents the Grad-CAM visualizations generated from three randomly selected jute leaf images from different categories, highlighting the most influential regions in the model’s decision-making process. These images are used to objectively evaluate the model’s effectiveness. Grad-CAM highlights the model’s ability to focus on the most relevant areas within an image, aiding in the accurate classification of different leaf types. The red and yellow regions in the visualizations indicate the key parts of the image that significantly influence the model’s predictions. The figure clearly shows that the model focused on diseased areas; notably, the regions highlighted by Grad-CAM closely align with the visibly affected parts of the leaves, demonstrating the model’s ability to distinguish and localize disease symptoms that were clearly observable to the human eye. The red bounding box drawn from the Grad-CAM heatmaps shows the diseased area more clearly, which enhanced the interpretability of the model by visually confirming that its predictions are based on the actual symptomatic regions present on the leaf. The visual evidence provided by Grad-CAM not only enhanced the interpretability of the model’s predictions but also reinforced its reliability for practical agricultural applications, where understanding the focus of diagnostic tools is crucial for ensuring effective disease management. **Figure 13** presents the Grad-CAM visualizations for images that were misclassified by the model. It indicates that the model focused on irrelevant or misleading regions of the image, which likely contributed to the incorrect predictions.

### 4.5 Web application results

**Figure 14:**
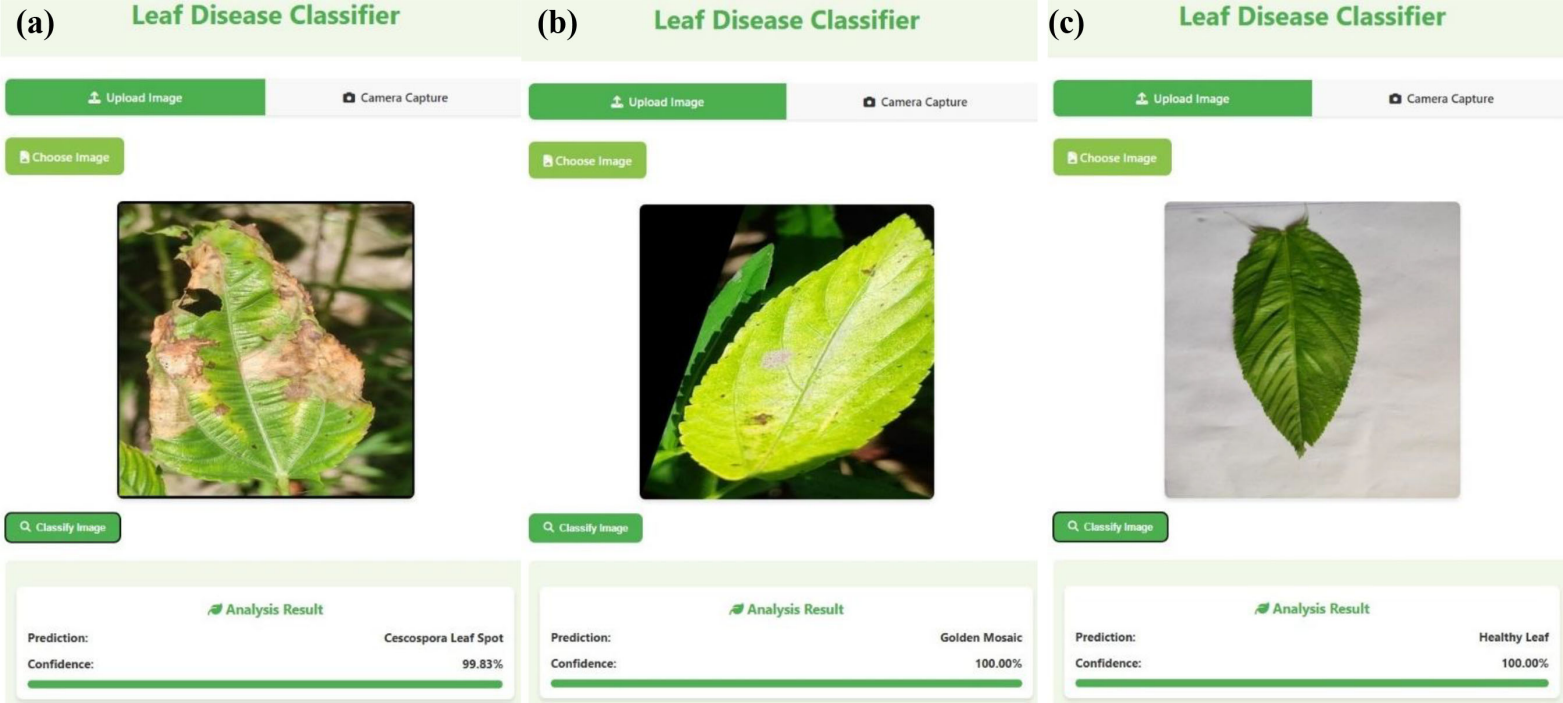
Screenshots from the deployed Flask-based web application demonstrating real-time classification results for jute leaves: **(a)** Cercospora leaf spot, **(b)** golden mosaic, **(c)** healthy leaf.

**Figure 14** illustrates the results obtained from the deployed web application, highlighting its ability to perform real-time plant disease detection through a user-friendly interface. The application allows users, particularly farmers, to upload images of jute leaves and receive immediate diagnostic feedback. This real-time interaction demonstrates the practical utility of the proposed system under field conditions, supporting timely decision-making and promoting accessible, technology-driven agricultural management.

The web application offers users the flexibility to either upload a preexisting image or capture one in real time via the device’s integrated camera. After selecting or capturing an image, users can initiate the classification process by clicking the “classify image” button, which activates the preloaded DL model to analyze the input and produce a prediction. This user-friendly interface enables smooth and effective evaluation of the model’s performance on both static and live image data.

## 5 Discussion

### 5.1 Comparative analysis

**Table 11** shows the comparative analysis between previous works on jute leaf disease classification and the method proposed in this study. It should be noted that only leaf diseases are compared here as jute pest and plant disease classifications are not within the scope of this work. The supervised learning method utilizing the full 80-10–10 data split yielded the highest performance, achieving an accuracy of 98.95%. In contrast, when only 10% of the labeled data were used in a supervised setting, the performance decreased significantly, with an accuracy of 89.47%, highlighting the limitations of supervised models in limited label scenarios. However, when the same 10% labeled data were used in combination with 90% unlabeled data through the proposed confidence regularization ST method, the model remarkably achieved 97.89% accuracy.

**Table 11:** Performance comparison of the proposed model with previous state-of-the-art models on jute leaf disease classification.

Reference	Data	Classes	Number of images in the dataset	Dataset availability	Method	Semi-supervised	Accuracy	Precision	Recall	Web app	XAI	Number of parameters (Million)
Bansal et al. (2024)	Federated learning data distributed among 5 geographical clients	anthracnose, stem rot, leaf mosaic pattern, macrophomina wilt effect, and blight	4,200	No	Federated CNN	No	94	83.90	83.89	No	No	473.65
Kaushik and Khurana (2025)	Traditional supervised 80–20 data split	Diseased and healthy	1,820	No	Fine-tuned ResNet50	No	94	94	94	No	No	24.77
Rajput et al. (2024)	Distributed federated learning among 5 clients	anthracnose, stem rot, root rot, Macrophomina Wilt, and yellow mosaic virus	4740	No	Federated CNN + Decision Tree	No	98	94.28	94.26	No	No	368.81
Uddin and Munsi (2023)	Supervised 80-12–8 data split	healthy, yellow mosaic, and powdery mildew	4140	No	Custom CNN	No	96	95.70	96.10	No	No	36.84
Tanny et al. (2025)	Supervised learning	Cescospora leaf spot, golden mosaic, and healthy leaf	920	Yes	DERIENet, a deep ensemble learning model with ResNet50, InceptionV3, EfficientNetB0	No	**99.95**	**99.89**	**99.89**	No	No	52.33
Proposed	Supervised 80-10–10 data split	Cescospora leaf spot, golden mosaic, and healthy leaf	920	Yes	Proposed custom CNN	No	98.95	98.99	99.07	**Yes**	**Yes**	**2.24**
Proposed	Supervised with 10% labeled data	Cescospora leaf spot, golden mosaic, and healthy leaf	920	Yes	Proposed custom CNN	No	89.47	90.20	90.28	**Yes**	**Yes**	**2.24**
Proposed	Semi-supervised (10:90 labeled and Unlabeled data ratio)	Cescospora leaf spot, golden mosaic, and healthy leaf	920	Yes	Proposed custom CNN with semi supervised self-training	**Yes**	**97.89**	**97.80**	**98.15**	**Yes**	**Yes**	**2.24**

Bold numbers and text indicate best results.

Recent studies on jute leaf disease detection demonstrate a range of methods, datasets, and outcomes. The accuracies of our supervised and semi-supervised models are better than or closely equal to all the past studies except Tanny et al. (2025) who used our dataset and produced a model with an accuracy of 1% better than our comparable supervised model and 2.05% better than the proposed semi supervised model. The model by Tanny et al. had much larger parameters (23 times) limiting its deployment opportunities in real world scenarios and no XAI was used to demonstrate the model was capable of correctly identifying the disease affected regions. Furthermore, they split the data in training, testing and validation sets after the augmentation, resulting in data leakage which could be the reason for the higher accuracy they obtained. On other hand, in this study, augmentation was carried out after the data split to avoid any data leakage.

A major limitation of the previous approaches was their reliance on large amounts of labeled data, which are often scarce in real-world scenarios. Semi-supervised learning employed in this study addressed this challenge by leveraging abundant unlabeled data alongside limited labeled examples to improve model performance. Also, the previous models had significantly large number of parameters, making them computationally heavier and less efficient for deployment in resource-constrained environments. Furthermore the jute leaf disease classes used in this study were not exactly the same classes available in the literature, which made it difficult to conduct a direct comparison even with three jute leaf disease classes (Uddin and Munsi, 2023; Haque et al., 2024).

### 5.2 Strengths, practical implication, limitations, and future work

The proposed custom CNN model demonstrated strong effectiveness in classifying jute leaf diseases using both supervised and semi-supervised learning approaches. Notably, under a semi-supervised framework utilizing only 10% labeled and 90% unlabeled data through a confidence regularization (ST) method, the model still achieved a high accuracy of 97.89%. The near-supervised performance demonstrated the strength of the semi-supervised approach in effectively leveraging unlabeled data to compensate for the scarcity of labeled examples. Overall, the analysis confirmed that the proposed model is not only capable of high-accuracy classification with limited data but also outperforms traditional methods by minimizing the dependency on costly labeled datasets, making it particularly advantageous for real-world agricultural applications. The model’s lightweight architecture comprising just 2.24 million parameters and occupying only 8.54 MB of memory made it highly suitable for deployment in resource-constrained environments such as rural farms with limited computational infrastructure. Integrated into a web application, the model allowed for real-time, accessible disease detection, empowering farmers to take timely action and implement smarter, data-driven crop management strategies. It also incorporated explainable AI (XAI) techniques, specifically Grad-CAM, to enhance interpretability by identifying regions of interest and accelerating disease localization critical for real-time field analysis. Moreover, the model is uniquely combined with a semi-supervised self-training framework and real-time web application development for jute leaf disease detection, aspects not jointly addressed in prior studies.

However, the use of custom layers in the proposed model such as grouped convolutions and SE blocks, may introduce some overhead in training time and deployment complexity on less optimized hardware. The model’s performance can also be influenced by environmental factors such as lighting, background variability, and image resolution, which may limit generalizability across different real-world conditions. Furthermore, while the model leveraged unlabeled data effectively, the quality of that data remained a crucial factor in sustaining classification performance. A major limitation of this study is that the proposed model was evaluated only on a self-collected jute leaf dataset. While the results demonstrate strong performance, additional validation on larger and more diverse crop disease datasets is needed to fully establish robustness and generalizability.

Future work could focus on improving robustness through increasing the classification accuracy, domain adaptation techniques to enhance generalization in varied environmental settings. Additionally, exploring self-supervised or active learning strategies may further reduce reliance on labeled data. Optimizing the model for real-time execution on edge devices would also enhance its practical deployment, enabling efficient AI-driven disease diagnosis directly in the field. Overall, this solution advanced the goals of sustainable farming by supporting early disease detection, minimizing yield loss, and contributing to more resilient, precision-driven agriculture. Furthermore, as part of future work, a structured usability study has been planned to conduct in three phases: (i) expert validation with agronomists to assess classification reliability, interface clarity, and consistency with field diagnosis; (ii) pilot testing with farmers in smallholder communities, where 20–30 participants will use the web application on mobile devices under real farming conditions to evaluate ease of use, clarity of results, and trust in the predictions; and (iii) field-scale evaluation, involving integration of user feedback to refine the interface, add local language support, and test robustness under diverse environmental conditions. Metrics such as task completion time, error rate, user satisfaction scores, and adoption intent will be recorded to ensure a comprehensive evaluation. This roadmap will provide actionable insights into end-user needs, thereby enhancing the application’s usability, accessibility, and practical adoption in real agricultural settings.

## 6 Conclusions

This study presents a lightweight custom CNN for the classification of jute leaf diseases using both supervised and SSL strategies. By incorporating grouped convolutions, modified depthwise separable convolutions, enhanced SE blocks, and MBconv layers, the model achieved high representational efficiency with only 2.24M parameters (8.54 MB). The proposed architecture attained a mean accuracy of 98.32% with precision, recall, and F1-scores all above 98%, demonstrating strong stability and robustness. In the supervised setup, the model achieved 98.95% accuracy, while the semi-supervised confidence regularization self-training approach achieved 97.89% accuracy with only 10% labeled and 90% unlabeled data, confirming the model’s capability to deliver near-supervised performance with minimal labeling effort. Beyond accuracy, the integration of Grad-CAM added interpretability by highlighting diseased regions, while the development of a Flask-based web application demonstrated practical field applicability. These aspects collectively bridge the gap between high-performing AI models and their real-world usability in agriculture.

The findings highlight three key contributions: (i) reducing reliance on costly labeled datasets through SSL, (ii) enabling deployment in resource-constrained environments via a lightweight architecture, and (iii) ensuring transparency and usability through explainable AI and a functional application. Nevertheless, the absence of usability testing with agronomists and farmers is a current limitation.

## Data Availability

Publicly available datasets were analyzed in this study. This data can be found here: https://www.kaggle.com/datasets/mdsaimunalam/jute-leaf-disease-detection.
